# Ontologies Applied in Clinical Decision Support System Rules: Systematic Review

**DOI:** 10.2196/43053

**Published:** 2023-01-19

**Authors:** Xia Jing, Hua Min, Yang Gong, Paul Biondich, David Robinson, Timothy Law, Christian Nohr, Arild Faxvaag, Lior Rennert, Nina Hubig, Ronald Gimbel

**Affiliations:** 1 Department of Public Health Sciences Clemson University Clemson, SC United States; 2 College of Public Health George Mason University Fairfax, VA United States; 3 School of Biomedical Informatics The University of Texas Health Sciences Center at Houston Houston, TX United States; 4 Clem McDonald Biomedical Informatics Center Regenstrief Institute Indianapolis, IN United States; 5 Loweswater Consulting Combria United Kingdom; 6 Ohio Musculoskeletal and Neurologic Institute Ohio University Athens, OH United States; 7 Department of Planning Aalborg University Aalborg Denmark; 8 Department of Neuromedicine and Movement Science Norwegian University of Science and Technology Trondheim Norway; 9 School of Computing Clemson University Clemson, SC United States

**Keywords:** clinical decision support system rules, clinical decision support systems, interoperability, ontology, Semantic Web technology

## Abstract

**Background:**

Clinical decision support systems (CDSSs) are important for the quality and safety of health care delivery. Although CDSS rules guide CDSS behavior, they are not routinely shared and reused.

**Objective:**

Ontologies have the potential to promote the reuse of CDSS rules. Therefore, we systematically screened the literature to elaborate on the current status of ontologies applied in CDSS rules, such as rule management, which uses captured CDSS rule usage data and user feedback data to tailor CDSS services to be more accurate, and maintenance, which updates CDSS rules. Through this systematic literature review, we aim to identify the frontiers of ontologies used in CDSS rules.

**Methods:**

The literature search was focused on the intersection of ontologies; clinical decision support; and rules in PubMed, the Association for Computing Machinery (ACM) Digital Library, and the Nursing & Allied Health Database. Grounded theory and PRISMA (Preferred Reporting Items for Systematic Reviews and Meta-Analyses) 2020 guidelines were followed. One author initiated the screening and literature review, while 2 authors validated the processes and results independently. The inclusion and exclusion criteria were developed and refined iteratively.

**Results:**

CDSSs were primarily used to manage chronic conditions, alerts for medication prescriptions, reminders for immunizations and preventive services, diagnoses, and treatment recommendations among 81 included publications. The CDSS rules were presented in Semantic Web Rule Language, Jess, or Jena formats. Despite the fact that ontologies have been used to provide medical knowledge, CDSS rules, and terminologies, they have not been used in CDSS rule management or to facilitate the reuse of CDSS rules.

**Conclusions:**

Ontologies have been used to organize and represent medical knowledge, controlled vocabularies, and the content of CDSS rules. So far, there has been little reuse of CDSS rules. More work is needed to improve the reusability and interoperability of CDSS rules. This review identified and described the ontologies that, despite their limitations, enable Semantic Web technologies and their applications in CDSS rules.

## Introduction

For more than half a century, clinical decision support systems (CDSSs) have been developed and used in clinical care delivery [1-5]. Some early CDSS examples include Dialog [6], INTERNIST-1 [7-9], Quick Medical Reference [8], and Iliad [10-12]. The effectiveness of CDSSs in clinical care has been established [13-15], with some pioneering researchers’ work on CDSS effectiveness particularly noteworthy [16]. Researchers have examined CDSS users’ and developers’ experiences, discussed their CDSS vision for the future [17], and recommended best practice guidelines in CDSSs [18-22]. Meanwhile, the challenges of CDSSs have been well documented [23]. Meeting clinician information needs is one way a CDSS can help health care providers improve clinical care quality. Many studies, such as Infobutton [13,24], have demonstrated the effectiveness of CDSSs in this aspect. CDSSs are currently routinely used in clinical care, with rates ranging from 68.5% to 100% in primary care settings based in offices [25] in the United States as part of electronic health record (EHR) systems. CDSSs can take many forms, including but not limited to reminders for preventive services (eg, immunizations and screening tests) [26-28], alerts for drug-drug interactions [22,29,30], diagnostic or treatment plan recommendations [31-33], clinician content assistance [34-38], and recommendations for adhering to current clinical practice guidelines [39-41]. CDSSs have played an important role and are widely used in practice to provide safer and better clinical care services.

CDSS rules, which function similarly to the human central nervous system, direct the behaviors of a CDSS during operations by incorporating patient data, contextual information, and medical domain knowledge. The central role of CDSS rules is a decisive factor in the relevance and usefulness of a CDSS in the overall clinical workflow, which impacts whether a CDSS is adopted and routinely used. CDSS rules can be written in Arden syntax [42], Semantic Web Rule Language (SWRL), Jess, Jena, and other programming languages, and the processes are labor intensive. Only specially trained personnel are qualified to write such rules. Moreover, regular updating of CDSS rules is required to keep CDSSs relevant and useful in clinical care delivery. However, the process of developing, updating, and maintaining CDSS rules is time-consuming and resource intensive [4,43], making it difficult for both large institutions and resource-constrained small-scale practices. CDSS rule usage data, such as rule fire rates, overwrite rates, successful rates, and user feedback data, can be collected to improve and customize CDSSs and manage CDSS rules. Typically, CDSS rule maintenance entails adding, deleting, and updating CDSS rules.

Ontologies have been successfully applied to generate and supply domain knowledge in the use, reuse, sharing, and interoperability of information. Ontologies are seen as promising solutions to the challenges of managing and maintaining CDSS rules across institutional boundaries. The Semantic Web is a technology enabled by ontology [44] that is critical in information sharing and reuse [45,46], medicine [47], and CDSSs [48,49]. Although there are numerous definitions of ontology, we used Gruber’s definition in this manuscript: “an ontology is a specification of conceptualization” [45]. Interoperability has been identified as a major challenge for health care information technologies, particularly when it comes to sharing health information across institutional or national boundaries. Ontologies have the potential to shorten the interoperability gap.

Reusing and sharing CDSS rules are important, but they are not yet routine operations; thus, we conducted this systematic literature review. This study aims to expand on the current state of using ontologies in CDSS rules by conducting a systematic review of the literature on the intersection of CDSS rules, Semantic Web technologies (particularly ontologies), and use of ontologies in CDSSs. The review is expected to provide a comprehensive view of using ontologies in CDSS rules, with granular details. The results could serve as a basis to form a knowledge framework of the topic that may inspire future research. The research question we intend to answer with this systematic literature review is as follows: What is the current state of using semantic technologies, particularly ontologies, to leverage CDSS rule interoperability? Furthermore, the manually annotated results of selected publications could serve as gold standards for automatically identifying relevant entities in the literature.

## Methods

### Databases and Search Strategies

**Figure 1:**
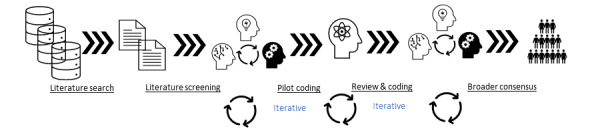
General workflow of the systematic literature review.

Figure 1 illustrates the general workflow we used to conduct this literature review. An initial set of literature searches was conducted on June 2, 2020, which was followed by a review and discussions. The reviewers (XJ, HM, and YG) refined and agreed with the search strategies and searched PubMed, the Association for Computing Machinery (ACM) Digital Library, and the Nursing & Allied Health Database (NAHD) for literature, using the search strategies mentioned below. A final search was conducted on January 5, 2022, in the 3 literature databases as an update.

For PubMed, the following search was conducted: (clinical decision support systems[MeSH Terms]) AND (ontolog*[Title/abstract] OR rule*[Title/abstract]). For ACM Digital Library, the following search was conducted within the scope of the ACM Guide to Computing Literature: [[Publication Title: “clinical decision support*”] OR [Publication Title: cds*]] AND [[Publication Title: ontolog*] OR [Abstract: ontolog*] OR [Publication Title: rule*] OR [Abstract: rule*]]. For the NAHD, the following search was limited to peer-reviewed publications: mesh(clinical decision support) AND (ti(ontology) OR ti(ontologies) OR ab(ontology) OR ab(ontologies) OR ti(rule) OR ti(rules) OR ab(rule) OR ab(rules)).

### Inclusion and Exclusion Criteria

The inclusion criteria were as follows: text was written in English; full-text publication was available; ontologies were designed to be implemented or were already implemented in CDSSs, particularly related to CDSS rules; content included the granularity of CDSS rules; ontologies were designed to be integrated or were already integrated with health information systems (eg, EHRs), either in a production system or a prototype, with at least one architecture diagram, applied in clinical domains or designed for clinical domains to support health care providers; the publication was peer-reviewed; and details on the integration of CDSSs and EHRs were present for evaluation studies.

The exclusion criteria were as follows: only CDSS rules were included, regardless of the stage of the CDSS rule lifecycle (ie, development, identification, refinement, validation, evaluation, or implementation) or there was no mention of integration or ontologies; only ontologies were developed, evaluated, and validated, or there was no mention of integration or a CDSS; the system was designed without mentioning the granularity of CDSS rules or ontologies; and nonclinical decisions, such as administrative or management decisions (eg, supply chain management), were described.

### General Workflow for Screening Papers

The first 100 papers were screened by all 3 authors (XJ, HM, and YG) independently. The first 100 retrieved papers were initially screened by 1 author (XJ) to draft initial inclusion and exclusion criteria. The inclusion and exclusion criteria were refined and adjusted by 2 authors (HM and YG) during the iterative screening, review, analysis, and discussions. Further, 2 authors (HM and YG) replicated the screening, and all 3 authors discussed and validated the results. The rest of the papers were then screened by at least 2 authors (XJ and HM, or XJ and YG) independently to determine inclusion. Disagreements were discussed and resolved via iterative rounds of group meetings.

The screening and manual review processes were conducted independently and approved by at least 2 authors. The literature was first screened based on titles, abstracts, and full-text publications when needed. The papers that were included were then manually coded to provide more content analysis and synthesized evidence. The final results were shared among all the authors. All disagreements were settled through group discussions.

### Reviewing, Coding, Analyzing, and Synthesizing Processes

We followed grounded theory during the reviewing and manual coding of the included publications. One author (XJ) randomly selected 10 papers from the included 81 papers to start the coding (annotating) based on the focus of this literature review. ATLAS.ti 9 (desktop and web versions; ATLAS.ti Scientific Software Development GmbH), a qualitative data analytic tool, was used for coding. The coding results were discussed by 3 authors (XJ, HM, and YG). The discussion results formed the first draft of codes and code groups (Multimedia Appendix 1), that is, data items. Three coders (XJ, HM, and YG) then reviewed and coded the first 40 of the included papers using the initial principles and code groups, and added new codes and code groups when needed. Then, a second set of meetings was used to obtain consensus on updated principles and code groups. Refined codes and code groups were used to code the remaining papers. Every paper was coded by at least 2 coders independently. The coding results were then compared, and any discrepancies were resolved by group discussions. The code groups and codes were revised, consolidated, and updated during each discussion. Multimedia Appendix 2 presents the refined code groups and examples. Data items emerged during the review and were refined via discussions instead of predefinition before reviewing. Multimedia Appendix 3 lists all included papers.

After coding, the literature was analyzed and synthesized with a focus on several aspects, including CDSS application domains, CDSS mechanisms used in clinical settings, CDSS rule formats, authoring, management, and the roles of ontologies. The 3 authors worked together in an iterative process of analysis and synthetization. After obtaining consensus among all 3 authors, the results were then shared and discussed among all authors. Any concerns, confusions, or disagreements among the authors were resolved through iterative discussions. We followed the PRISMA (Preferred Reporting Items for Systematic Reviews and Meta-Analyses) 2020 checklist [50] for reporting the systematic review with all relevant items (Multimedia Appendix 4 and Multimedia Appendix 5).

## Results

### Overview

**Figure 2:**
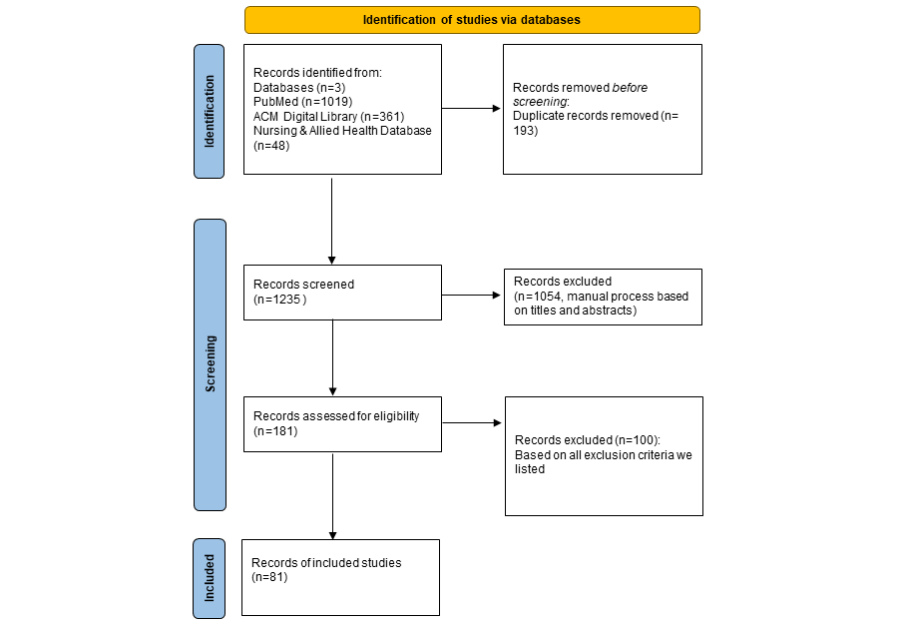
Flowchart of the literature search, screening, and selection. ACM: Association for Computing Machinery.

**Figure 3:**
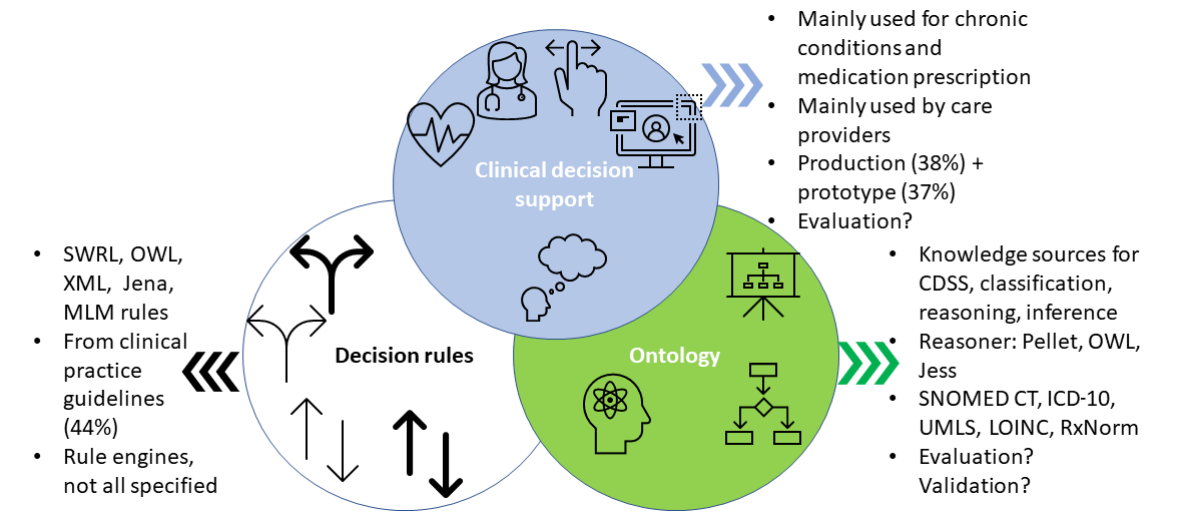
Initial knowledge framework on clinical decision support systems (CDSSs), CDSS rules, and ontology applications in CDSSs. ICD-10: International Statistical Classification of Diseases and Related Health Problems, 10th Revision; LOINC: Logical Observation Identifiers Names and Codes; MLM: medical logic module; OWL: Web Ontology Language; SNOMED CT: Systematized Nomenclature of Medicine-Clinical Terms; SWRL: Semantic Web Rule Language; UMLS: Unified Medical Language System; XML: Extensive Markup Language.

By January 5, 2022, literature searches retrieved 1235 publications from 3 sources. After removing duplicates and examining according to the inclusion and exclusion criteria, 81 publications (Multimedia Appendix 3) were included in the final review and analysis [26,27,29,31-33,51-125]. Figure 2 depicts the literature search, screening, selection flow, and results. Figure 3 summarizes the main components covered by the literature review and the summary findings, and serves as an initial knowledge framework on CDSSs, CDSS rules, and ontology applications in CDSSs.

The majority of the publications (73/81, 90%) included in the review were from PubMed, a dominant source. After removing duplicates, the ACM Library added 8 new publications. After cleaning, discussion, and consolidation, 30 code groups and 221 final codes were used in ATLAS.ti (Multimedia Appendix 2). These codes and code groups guided our analysis and synthesis of the results. Multimedia Appendix 6 shows a word cloud image generated by ATLAS.ti that reflects the codes coded in the publications included.

PRISMA 2020 is designed to guide the reporting of outcome-oriented studies. Our systematic literature review focused on the design, development, and implementation of CDSSs, particularly related to CDSS rules and ontologies. Therefore, effect measures or certainty assessments were irrelevant items. We reported 19 categorical items (out of 27 categorical items, 26 items out of 42 items; Multimedia Appendix 4) for the full-text papers and 10 for the abstracts (out of 12 items; Multimedia Appendix 5).

### Results Related to CDSS Characteristics

Over one-third (29/81, 36%) of CDSSs were designed and used for chronic condition management, prediction, or risk assessment, including but not limited to type 1 and 2 diabetes, hypertension, and asthma. Medication prescriptions (13/81, 16%), such as medication ordering, detection of adverse drug events, drug-drug interactions, and cancer care (8/81, 10%), were also significant application domains. Multimedia Appendix 7 illustrates the clinical domains of CDSSs within the included publications. Most CDSSs were designed for health care providers, but only 11% (9/81) were intended for patients. Most CDSSs provided recommendations, suggestions, alerts, or reminders. Among all the items in our comparison (Multimedia Appendix 8), EHR evaluation studies within the operational systems or prototypes exhibited the least complete information. Evaluations of CDSSs have been listed in multiple columns in Multimedia Appendix 8. Some CDSSs were implemented in production systems (31/81, 38%), whereas others were implemented in prototypes (30/81, 37%), which included experimental systems. Multimedia Appendix 8 summarizes the key features of CDSSs identified in the publications. In all tables, we adopted the original terms used in the corresponding papers. Some papers, for example, referred to “physicians” as CDSS users, whereas others referred to “clinicians” as CDSS users.

### Results Related to CDSS Rules

Most CDSS rules were written in Web Ontology Language (OWL; 11/81, 14%), Extensive Markup Language (XML; 10/81, 12%), SWRL (9/81, 11%), Jena rules (5/81, 6%), and medical logic module (MLM; 3/81, 4%). Moreover, 2 publications [117,119] used N3 Language and 2 [90,117] used Natural Rule Language (NRL). Multimedia Appendix 9 presents 54 publications with more details on the CDSS rules, that is, publications that can fill out 3 or more cells (except for authors and publication year).

The most significant CDSS rule source is from clinical practice guidelines (36/81, 44%). Other sources of CDSS rules included domain expert input, publications (eg, textbooks and papers), multimedia sources, and internet resources. Data mining results were involved in CDSS rule sources [67,73]. CDSS rule authoring and editing tools were not routinely specified in the publications. Protégé [115] was the most prevalent tool to edit and author CDSS rules. Several publications also described developing authoring and editing tools [57,65,91].

There was a lack of technical details regarding rule engines, among which Jena (6/81, 7%), inference engine (6/81, 7%), Jess (4/81, 5%), JBoss (3/81, 4%), guideline engine (3/81, 4%), Drools (2/81, 3%), and Bayes (2/81, 3%) were frequently mentioned. Multimedia Appendix 9 summarizes how the CDSS rule (operation) works in a simplified manner. Many publications did not specify the working mechanism of CDSS rules within the EHR, electronic medical record (EMR), or hospital information system (HIS) context.

The majority of the publications did not appear to be focused on interoperability. Few papers that discussed interoperability (Multimedia Appendix 9) used HL7 CDA (Health Level 7, Clinical Document Architecture) or HL7 FHIR (Health Level 7, Fast Healthcare Interoperability Resources) standards. However, it is worth noting that such HL7 measures were not specifically designed for CDSS rules but rather for CDSS input and output.

Furthermore, some publications lacked necessary information for explaining the mechanisms of the systems, which can be critical barriers to reproducibility. Some publications lacked critical information, such as CDSS architecture diagrams; CDSS rule engines; CDSS rule languages; backend management methods for CDSS rules; and integration mechanisms among CDSS rules, ontologies, and EHR, EMR, or HIS systems.

### Results Related to Ontologies

In the included publications, ontologies were primarily used as knowledge sources for CDSSs (32/81, 40%) to facilitate classification (7/81, 9%), reasoning, and inference (6/81, 7%; eg, identification recommendations or relationships). Moreover, ontologies were used to specify CDSS rules (12/81, 15%) or to provide general knowledge for the EMR or EHR systems. These 2 applications overlapped in some cases (19/81, 24%; ie, the ontologies were used to provide specified CDSS rules and general knowledge).

In the included publications, the terms “reasoner” and “rule engines” were used interchangeably. *Reasoner*, in our opinion, refers to the inference for a consistency check or classification for an ontology. A reasoner can be part of an ontology tool or can be external. For CDSSs, a *rule engine* is the mechanism that generates or provides recommendations by incorporating a patient’s data, contextual information, and medical knowledge (typically from an ontology or knowledge base). However, we kept the authors’ choice of terms in tables without modification. Among the included publications, the most common reasoners were Pellet (11/81, 14%), Jena (4/81, 5%), OWL reasoner (3/81, 4%), Jess (2/81, 3%), and the Euler/EYE inference engine (2/81, 3%).

The content and code systems used to represent the content should be included as ontology sources. The content could come from a popular textbook or a clinical practice guideline. The content can be coded in a specific code system, such as SNOMED CT (Systematized Nomenclature of Medicine-Clinical Terms). Multimedia Appendix 10 includes code systems that served as ontology sources. The most often used coding systems among the included publications were SNOMED CT (9/81, 11%), the International Statistical Classification of Diseases and Related Health Problems, 10th Revision (ICD-10; 4/81, 5%), Unified Medical Language System (UMLS; 4/81, 5%), Logical Observation Identifiers Names and Codes (LOINC; 3/81, 4%), and RxNorm (2/81, 3%).

Incomplete information for ontology validation is a common issue shown in the literature. Approximately 20 publications mentioned some validation, including validation or evaluation by domain experts (20/81, 25%). Some ontologies were authored by domain experts [55,63]. Multimedia Appendix 10 provides more information on the roles of ontologies in publications, including publications with 3 or more cells (except for authors and publication year; n=36).

## Discussion

### Summary of the Results

Although ontologies contribute to the content of CDSS rules and have the potential to facilitate interoperable CDSS rules, our systematic review showed that reusing and sharing of CDSS rules have not been achieved. CDSSs have a wide range of clinical application domains, primarily for health care providers, such as chronic condition management, medication ordering, and cancer care. CDSS rules are primarily based on clinical practice guidelines.

Although reusing and sharing CDSS artifacts are well-recognized challenges [1,109], reusability, customization, and shareability of CDSS rules are not yet a common focus, even in publications focusing on CDSS rule editing [43,126,127]. These are important topics to cover in a literature review. Marco-Ruiz et al [109] demonstrated how to use CDSS artifacts in the Linked Data framework [128] by leveraging Semantic Web technologies, particularly ontology. However, that work was at a higher level, describing concepts without tangible tools implemented in clinical practice. To fill this gap, one approach is to build an upper-level CDSS ontology [129] to encourage the reuse of CDSS rules and demonstrate the potential of ontologies. Our effort is in alignment with their vision, as well as other efforts in reusing and sharing CDSS artifacts [1,109].

Ontologies were not at the center of any early examples of CDSSs [6-12]. An early demonstration of using medical terminology in CDSSs was the adoption of Current Medical Information and Terminology (CMIT) in a diagnostic engine [130,131]. Even under our “loose use of ontology” during our systematic literature search, there was no case in which ontology played a central role in sharing CDSS rules, particularly for rule management and maintenance.

Over the years, CDSSs have been successfully applied in clinical care. Unfortunately, CDSS rules are not yet portable. Making CDSS rules more portable is therefore significant work that could be leveraged by ontologies, and our systematic literature review brings us one step closer to that goal. Marco-Ruiz et al also conducted a very relevant systematic literature review. However, their focus was on the interoperability mechanisms used in CDSSs [132,133]. According to the results of their systematic literature review, 32% of the included papers used ontologies and 46% used standard terminologies. The findings related to ontologies are similar [132,133] to those of our paper. However, we presented a more detailed and thorough analysis of these technologies used in CDSS rules. Nevertheless, both papers concluded that complete CDSS interoperability is not a reality. Thus, additional efforts are required to achieve interoperable and reusable CDSS artifacts, such as CDSS rules.

### Interpretation of the Results

Rule engines, which execute rules, patient data, and context information to produce a result, such as an alert or a recommendation, are critical components of CDSSs [1]. Jess, a rule engine and development environment in Java [134], was frequently mentioned in the included publications as a tool for developing rule-based CDSSs. SWRL rules can be converted to Jess rules in the popular tool Protégé, using a plug-in application programming interface (API) SWRLJessTab. Jess rules can be used by the Jess rule engine, which is widely used in rule-based expert systems [134]. In addition to Jess, Jena and Drools were used frequently in the publications included. Jena is a Java API that supports rule-based inference and makes use of resource description framework (RDF) graphs [135]. Jena.java API is a popular framework for managing RDF/OWL descriptions and can handle OWL models [96]. Drools is a business rule management system that includes a rule engine [136]. Drools also has the SWRL API that supports SWRL and Semantic Query-Enhanced Web Rule Language (SQWRL). SWRL can be queried by SQWRL.

Reasoning via a reasoner is a critical characteristic of many ontologies, even though the current reasoning is still in first-degree logic. Reasoning can be used for the following 3 main functions: consistency check, classification, and realization [137]. Several publications specified the classification roles of the ontologies and reasoners (Multimedia Appendix 10). The Manchester University OWL group has curated an updated list of OWL reasoners [137]. Parsia et al [137] compiled and compared the current OWL reasoners and their performances via the competition report. Both Pellet and Jena are popular reasoners (Multimedia Appendix 10), and other reasoners include FaCT++ [98], Z3 Solver reasoner [105], Euler/EYE inference engine [117,119], OWL Horst [109], and OWL Cerebra [63] among the included publications. Among these reasoners, Pellet [138] is Java based, and it can work on SWRL rules and ontologies written in OWL2. SWRL was initially designed as a rule language for Semantic Web technologies [139]. A user needs the rule language and an editor (eg, Protégé SWRL tab) to write, revise, and query the rules. SWRL can be queried by SQWRL (a query language for OWL) or SPARQL (SPARQL Protocol and RDF query language). Reasoners can then be used to conduct reasoning based on the rules and facts defined in the ontology or knowledge base. Protégé-OWL [140] provides an editor for SWRL rules. Protégé SWRL editor is another example.

This review has demonstrated unique insights about CDSS rules, ontologies, and ontology applications, particularly in CDSS rule management and maintenance, and has presented several distinct characteristics that complement the existing literature. An earlier review [40] focused on clinical decision-making in forming ontologies to support complex cognitive processes and reasoning processes comparing evaluation metrics but did not cover the implementation of EHR, EMR, or HIS systems and the mechanisms of these characteristics.

### Significance of the Work

Our systematic review demonstrated the state-of-the-art applications of ontologies in CDSS rules. These applications have a lot of potential for reusing and sharing CDSS artifacts. However, none of the existing papers elaborated or demonstrated how ontologies enable portable CDSS rules. Although some authors recognized this benefit [1,43,109], none have conducted a systematic review. Our literature review thoroughly examined the topic, outlined the current frontlines on CDSS rules and ontology uses in CDSSs, established the knowledge framework, and compiled a comprehensive collection of relevant publications that can inform future efforts to design or improve CDSSs. This systematic review focused on the mechanisms of CDSSs in clinical practices or prototypes, CDSS rules, and ontology roles in CDSSs. The detailed information provided in each included publication (Multimedia Appendix 8, Multimedia Appendix 9, and Multimedia Appendix 10) about the reasoners, rule engines, ontologies, and CDSS rule formats used provided valuable references for designing or improving systems. The side-by-side comparison of publications (Multimedia Appendix 8, Multimedia Appendix 9, and Multimedia Appendix 10) also provided structured guidance for preparing future designs and publications or teaching references on the topics in tangible ways.

### Missing Information in the Publications and Our Recommendations

Inconsistent or missing information about CDSS rule languages, CDSS rule engines, and CDSS evaluation details was identified. In CDSS evaluation, there was commonly no information about how the evaluation was conducted or who performed the evaluation. There were also inconsistencies in technical details related to ontology purposes, reasoners, connection mechanisms, or communications between CDSSs and EHR, EMR, or HIS systems. Inconsistent or missing information hampered reproducibility and further improvement of published work. We are obviously not the only group that has identified missing critical information as a problem in technical papers on similar topics [141].

Another missing piece is the evaluation and validation of ontologies or knowledge bases. Only 25% of publications mentioned that domain experts conducted evaluation or validation. A formal assessment or validation is critical to ensure the validity of the results from automated processes for some ontologies (or knowledge bases) derived from other automatic methods (eg, machine learning algorithms). Testing has not been conducted consistently across the publications. Some ontologies were authored by domain experts, which provides greater validity than those involving nondomain experts while constructing ontologies.

Thus, it is recommended that authors include essential technical details in publications. These technical details include CDSS application domains, intended CDSS users, CDSS notification types, CDSS evaluations (what, how, and by whom), CDSS rule sources, CDSS rule languages, CDSS rule engines, CDSS operation mechanisms, ontology use purposes, ontology sources (both content and code systems), ontology validation, reasoners, and connection or communication mechanisms between CDSSs and EMR, EHR, or HIS systems. Authors are highly encouraged to include such details to help readers reference, compare, and increase the reproducibility of the reported work.

### Limitations

Our review has limitations. Non-English publications or full-text unavailable publications were not included. Publications that focused only on CDSS rules [43,126,127] were also excluded. Moreover, publications without specifying an ontology component were excluded, although such publications had a similar focus to one aspect of our systematic review. We also noticed that most of the publications on CDSS rule authoring and managing tools were from Partners HealthCare/Harvard Medical School. The strengths of Partners HealthCare/Harvard Medical School were shown. On the other hand, a lack of broad adoption, implementation, or publication of such topics was shown.

When “CDSS” is not specified as a keyword, the search results may exclude publications. For example, our 2 previous papers [142,143] were not found via the search strategy because “CDSS” was not used as a keyword, although the content was undoubtedly within the scope of this review. This challenge is common to how our current literature databases are organized and how we conduct a literature search. Even with MeSH (Medical Subject Headings; the controlled vocabulary for PubMed), publications can still be missed without using commonly recognized keywords. This challenge could be minimized and mitigated by carefully developing an exhaustive list of keywords to maximize the possibilities found during a literature search in the future.

### Conclusions

The reuse, management, and maintenance of CDSS rules are critical yet challenging for their clinical application. Although ontologies have been used to contribute to the content of CDSS rules, they have not been used to facilitate CDSS rule reuse and sharing. Building a CDSS ontology, which could be the first tangible step, requires bridging high-level visions and operational efforts. Semantic interoperability remains a major challenge that must be overcome to achieve reuse of CDSS artifacts, including CDSS rules. The realization of semantic interoperability will not only allow for the reuse of CDSS artifacts, which are resource intensive to develop and maintain, but also provide practical insights to achieve interoperable patient records. This has been a long-lost aspect, and health care providers will be able to access patients’ complete records to provide safer and higher quality care every time to every patient. We believe that making CDSS rules interoperable can provide insightful guidance for interoperable patient records.

Incomplete technical details on CDSS rules and ontologies presented in publications should be addressed in future publications by including more detailed information about architectural diagrams; the mechanisms of connection among ontologies, CDSS rules, and EHR, EMR, or HIS systems; CDSS rule languages; reasoners; rule engines; the validation or authorization of ontologies and CDSS rules; the purposes of ontologies; ontology sources; and the management and maintenance of CDSS rules. Such information can help researchers to optimize design and development while also increasing reproducibility. Finally, the knowledge framework and the summarization of included publications are expected to guide future CDSS improvements and innovations, CDSS rules, and the integration and communication of CDSSs with EHR, EMR, or HIS systems.

## Appendix

Multimedia Appendix 1 Initial draft of codes and code groups used in reviewing/coding.

Multimedia Appendix 2 Refined version of codes and code groups used in reviewing/coding.

Multimedia Appendix 3 List of papers included in this systematic literature review (n=81).

Multimedia Appendix 4 PRISMA 2020 item checklist reported in our systematic literature review.

Multimedia Appendix 5 PRISMA 2020 for Abstracts checklist reported in our systematic literature review.

Multimedia Appendix 6 Word cloud generated from ATLAS.ti based on codes in the included publications .

Multimedia Appendix 7 Bar chart generated from ATLAS.ti showing the clinical domains of clinical decision support systems in included publications.

Multimedia Appendix 8 Basic clinical decision support system profiles in included publications (n=81).

Multimedia Appendix 9 Comparison of clinical decision support system rule characteristics in included publications (n=54).

Multimedia Appendix 10 Comparison of ontology roles in included publications (n=36).

## Abbreviations

ACM: Association for Computing Machinery
API: application programming interface
CDSS: clinical decision support system
EHR: electronic health record
EMR: electronic medical record
HIS: hospital information system
HL7: Health Level 7
NAHD: Nursing & Allied Health Database
OWL: Web Ontology Language
PRISMA: Preferred Reporting Items for Systematic Reviews and Meta-Analyses
RDF: resource description framework
SNOMED CT: Systematized Nomenclature of Medicine-Clinical Terms
SQWRL: Semantic Query-Enhanced Web Rule Language
SWRL: Semantic Web Rule Language

## Data Availability

A request for additional data in addition to the results and appendices of this manuscript can be made to the corresponding author, and the final decision on data release will be made on a case-by-case basis, as appropriate.
