# Supplementary material for: Ontologies Applied in Clinical Decision Support System Rules: Systematic Review
Source: JMIR Med Inform. 2023 Jan 19;11:e43053. doi: 10.2196/43053 (PMC9896360; doi:10.2196/43053)
Supplement: Multimedia Appendix 1 [file medinform_v11i1e43053_app1.pdf]

Appendix 1 Initial draft of codes and code groups used in reviewing/coding

name	comment	codegroup 1	codegroup 2
Architecture diagram		Architeture design	
Author	Maybe we can start from the family name of the first author to record each paper.		
BFO		Ontology Sources	
Clinicians	Can be the evaluators	Evaluation	
DO		Ontology Sources	
Drools	Workflow engine-Drools-flow	Rule Engine	
DrugBank		Ontology Sources	
Expert		Ontology Sources	
Feasibility		Evaluation	
Galen Ontology		Ontology Sources	
GEM execution engine		Rule Engine	
Guidelines		Ontology Sources	
HPO		Ontology Sources	
ICD-10		Ontology Sources	
IDO		Ontology Sources	
Inference engine		Rule Engine	
JENA inference engine	Jena reasoner	Rule Engine	
JENA rule engine		Rule Engine	
JENA rules		CDSS Rule Format	
Jess reasoner		Rule Engine	
Jess Rule Engine		Rule Engine	
Jess rules		CDSS Rule Format	
JRules OWL plug-in		Rule Management	
Literature		Ontology Sources	
LOINC		Ontology Sources	
Medical records	Also can be patient records, patient cases, can be identified or de-identified	Ontology Sources	Evaluation
NCBI taxonomy		Ontology Sources	
NCI Terminology		Ontology Sources	
Ontology inference performa		Evaluation	

OWL		CDSS Rule Format	Ontology Language
Pattern matching	?	Rule Engine	
Production system		Architeture design	
Protege	It can be used as a visual editing tool for OWL rules	Rule Management	
Prototype		Architeture design	
Protégé SWRL tab for editing		Rule Management	
Protégé+ SWRL Jess Tab		Rule Management	
Publication year	This is also used to record the paper.		
Reasoner: COSI (Closed Worl		Rule Engine	
Rule authoring interface		Rule Management	
Rule-based reasoner	(e.g., Pellet)	Rule Engine	
SNOMED CT		Ontology Sources	
SWAN		Ontology Sources	
SWRL rules		CDSS Rule Format	
UMLS		Ontology Sources	
Use cases	Can be ICU or other units; also called scenarios.	Evaluation	
Web resources		Ontology Sources	
