# Supplementary material for: Ontologies Applied in Clinical Decision Support System Rules: Systematic Review
Source: JMIR Med Inform. 2023 Jan 19;11:e43053. doi: 10.2196/43053 (PMC9896360; doi:10.2196/43053)
Supplement: Multimedia Appendix 2 [file medinform_v11i1e43053_app2.pdf]

Appendix 2 Refined version of codes and code groups used in reviewing/coding

Codebook-literature review		
**Code**	**Comment**	**Code Groups**
Action variables		
activating devices		
Acute pain		
adverse drug event		
Adverse drug events detection		CDSS Purpose-Domain<br>Ontology Purpose<br>CDSS Purpose-Type
alarm		
Alerts	Can be drug-drug interactions, or drug-dosage or other types of alerts	Ontology Purpose<br>CDSS Purpose-Type
Alzheimer’s disease (AD)		CDSS Purpose-Domain
Antibiotic prescribing		CDSS Purpose-Domain<br>Ontology Purpose<br>CDSS Purpose-Type
Archetypes		
Architecture diagram		Architeture Design
Arden syntax		CDSS Rule Format
Asthma		
Atrial fibrillation		
Author	Maybe we can start from the family name of the first author to record each paper.	
Bayes engine		
BFO		Ontology Sources
Blood transfusion		
BRAIN rule engine		
Breast cancer		CDSS Purpose-Domain
Business Process Execution Language (BPEL)		
business logic		
business rules engine		
Business rules-ILOG		Rule Management<br>CDSS Rule Format
C++		
Cancer		
Care management	Including patient management, disease management	CDSS Purpose-Domain<br>Ontology Purpose
CCD		
CDA		

Chronic diseases		
Chronic heart failure		
Chronic kidney disease		
Classifier	Including to classify temporal patterns, to categorize patients into groups	Ontology Purpose
clinical pathways		
Clinicians	Can be the evaluators	CDSS Rule Sources<br>CDSS Evaluation (who, what, how, where)
CLIPS ruler sets		
Clubfeet deformities		
consultation document		
COPD		
Coronary artery disease		
CPG rule syntax		
de facto standard		
Decision variables		
Diabetes		CDSS Purpose-Domain
Diagnosis	It ca nbe recommendation or assistance	CDSS Purpose-Domain<br>Ontology Purpose
Diarrhea management		
Disease management		
DO		Ontology Sources
domain		
Domain experts		
Drools	Workflow engine-Drools-flow	Rule Engine
DrugBank		Ontology Sources
drug-drug interaction		
Dublin core		
dyslipidemia		
EHIR rule engine		
Emergency department		
Emergency room	Or emergencey department	CDSS Purpose-Domain
Euler/EYE inference engine		
evaluation		
Expert		CDSS Rule Sources<br>Ontology Sources
expert system		
FaCTþþsemantic reasoner		
Fall prevention		

Feasibility		CDSS Evaluation (who, what, how, where)
Feasibility study		
FHIR		
Follow up care		CDSS Purpose-Domain
Foundational Model of Anatomy (FMA		
Framework		
Framework|		
FSM(Finite State Machine) based execution engine		
Fuzzy logic inference		
Fuzzy rules		
Galen Ontology		Ontology Sources
Gastric cancer		
Gastroenterologist		
GELLO		
GEM execution engine		Rule Engine
GEM: the Guideline Elements Model		CDSS Rule Sources
General practitioner		
Guidelines		CDSS Rule Sources<br>Ontology Sources
Guidelines in GLIF3		CDSS Rule Sources
Head trauma		
Heart failure		CDSS Purpose-Domain
Hemodialysis		
HL7		
HPO		Ontology Sources
Hypertension		CDSS Purpose-Domain
ICD-10		Ontology Sources
ICD-9-CM		
ICU		CDSS Purpose-Domain
IDO		Ontology Sources
ILOG JRules		
Images		
implementation		
Infectious disease		CDSS Purpose-Domain
Inference engine		Rule Engine
inference rule		

Inference/reasoning	It can include recommendations, to get implicit relationships, assessment, Infer context information, patient conditions; Infer clinical tests on patients to detect AD	CDSS Rule Sources<br>Ontology Purpose
inference-based		
inpatient		CDSS Purpose-Domain
JADE		
Java		
JBoss rule		
Jena		
JENA inference engine	Jena reasoner	Rule Engine
Jena Reasoner		
JENA rule engine		Rule Engine
JENA rules		CDSS Rule Format
Jess reasoner		Rule Engine
Jess Rule Engine		Rule Engine
Jess rules		CDSS Rule Format
JRules	including Jrules OWL plug-in	Rule Management<br>CDSS Rule Author/Edit
JRules OWL plug-in		
JSON		
Knowledge source	It can incldue CDSS rule source; Derive data, support, interface, configuration models	Ontology Purpose
Lab		
language		
Lipid management		CDSS Purpose-Domain
Literature		CDSS Rule Sources<br>Ontology Sources
logic element (class)		
LOINC		Ontology Sources
lung cancer		
Medical records	Also can be patient records, patient cases, can be identified or de-identified	CDSS Rule Sources<br>Ontology Sources<br>CDSS Evaluation (who, what, how, where)
medication order		

MLM		
Natural Rule Language (NRL)		
NCBI taxonomy		Ontology Sources
NCI Terminology		Ontology Sources
Nehprology		
Neonatal		
Neurosurgery		
NICE guideline		CDSS Rule Sources
notification		
Object Contraint Language (OCL)		
Object Management Group (OMG)		
Oncology		
ontology		
Ontology inference performance		CDSS Evaluation (who, what, how, where)
Ontology is validated by domain experts		Ontology Validation By
OPCS		
operation rule		
Operational risk managment		
Outpatient		CDSS Purpose-Domain
OWL		Ontology Language<br>CDSS Rule Format
patient		
patient data		
patient input		
patient management		
patient monitoring		
patient safety		
Patients' records	This is for CDSS Evaluation that conducted via using patient's records retrospectively.	CDSS Rule Sources<br>CDSS Evaluation (who, what, how, where)
Pattern matching	?	Rule Engine
Pediatric		
Pediatric orthopaedist		
Pellet reasoner		
pneumonia		
Polypharmacy prescription		CDSS Purpose-Domain<br>Ontology Purpose<br>CDSS Purpose-Type
Postoperative managment		CDSS Purpose-Domain
Postoperative reports		

Prediction models/rules	Alvarado Score can be an example; also should include prediction models	CDSS Purpose-Domain<br>CDSS Purpose-Type
Preoperative		
Preoperative assessment		
Preoperative risk assessment		CDSS Purpose-Domain
Prescription		
Primary care providers	PCP can be a user group; can be knowledge source; can be an evaluator; can validate clinical knowledge.	CDSS Purpose-Domain
Production system	Including EMR, HIS, or EHR	Architeture Design
PROforma-based authoring		
Prognosis		
Prostatectomy		
Protege	It can be used as a visual editing tool for OWL rules; including Protégé SWRL tab, Protégé + SWRLJessTab,	Rule Management<br>CDSS Rule Author/Edit
Prototype		Architeture Design
provider input		
Publication year	This is also used to record the paper.	
purpose		
RacerPro reasoner		
RDF/JSON		
Reasoner: COSI (Closed World OWL Interpreter)		Rule Engine
Recommendations	It can be recommendations for care managment, diagnosis, treatment or progrnosis	Ontology Purpose<br>CDSS Purpose-Type

Reminder	It can be screening reminder, immunization, or other preventive service reminder	Ontology Purpose<br>CDSS Purpose-Type
Risk assessment		
risk management		
Rule authoring interface		Rule Management
rule engine JSR-94		
Rule-based reasoner	(e.g., Pellet)	Rule Engine
RxNorm		
SAGE		
Sage format		
SAGE model		
SEBASTIAN rules		
Self-managment		
sepsis		
Services on clinical data repository	This is a code for architecture mechanism for CDSS	Architeture Design
Simulated clinical setting for testing		
SNOMED CT		Ontology Sources
SPARQL		
Speech language therapy		
statistics-based		
Suggestion		
Surgery		
SWAN		Ontology Sources
SWRL rules		CDSS Rule Format
Telemedicine system		CDSS Purpose-Domain
temporal model		
Test results-lab		
tool		
Training system		
Treatment	It can be a recommendation or assistance	CDSS Purpose-Domain<br>Ontology Purpose
triage		
uEngine		
UMLS		Ontology Sources
Urology		
Use cases	Can be ICU or other units; also called scenarios.	CDSS Evaluation (who, what, how, where)
validation		
verification		

Warning		
WDSL		
Web resources		CDSS Rule Sources<br>Ontology Sources
Web services	Including Web API	Architeture Design
xml		CDSS Rule Format

Code Groups

Architecture Design (diagram, description)
Architeture Design
CDSS Evaluation (who, what, how, where)
CDSS Purpose
CDSS Purpose-Domain
CDSS Purpose-Perioperative
CDSS Purpose-Type
CDSS Purpose-User
CDSS Rule
CDSS Rule Author/Edit
CDSS Rule Engine
CDSS Rule Format
CDSS Rule Management
CDSS Rule Sources
CDSS Rule Sources/Ontology Sources

CDSS Setting
Guideline Engine
Integrated Development Environment (IDE)
Language
Metadata
Ontology Evaluation
Ontology Language
Ontology Purpose
Ontology Sources
Ontology Validation By
Program Language
Reasoner
Rule Engine
Rule Management
Standards
