# Supplementary material for: Ontologies Applied in Clinical Decision Support System Rules: Systematic Review
Source: JMIR Med Inform. 2023 Jan 19;11:e43053. doi: 10.2196/43053 (PMC9896360; doi:10.2196/43053)
Supplement: Multimedia Appendix 3 [file medinform_v11i1e43053_app3.pdf]

Appendix 3: List of selected papers included in this systematic literature review (*n* = 81)

Publishing year	Author	Paper title	Journal/source	PMID
2000	A Manjarrés Riesco, R Martínez Tomás, J Mira Mira	A customisable framework for the assessment of therapies in the solution of therapy decision tasks	Artif Intell Med. 2000 Jan;18(1):57-82	10606794
2000	S S Abidi, S Manickam	Transforming XML-based electronic patient records for use in medical case based reasoning systems	Stud Health Technol Inform. 2000;77:709-13	11187645
2000	De Clercq PA, Blom JA, Hasman A, Korsten HH	GASTON: an architecture for the acquisition and execution of clinical guideline-application tasks	Med Inform Internet Med. 2000 Oct-Dec;25(4):247-63	11198187
2000	Payne TH, Savarino J, Marshall R, Hoey CT	Use of a clinical event monitor to prevent and detect medication errors	Proc AMIA Symp. 2000:640-4	11079962
2001	Achour SL, Dojat M, Rieux C, Bierling P, Lepage E	A UMLS-based knowledge acquisition tool for rule-based clinical decision support system development	J Am Med Inform Assoc. 2001 Jul-Aug;8(4):351-60	11418542
2001	Séroussi B, Bouaud J, Dréau H, Falcoff H, Riou C, Joubert M, Simon C, Simon G, Venot A	ASTI: a guideline-based drug-ordering system for primary care	Stud Health Technol Inform. 2001;84(Pt 1):528-32	11604796
2002	Karadimas HC, Chailloleau C, Hemery F, Simonnet J, Lepage E	Arden/J: an architecture for MLM execution on the Java platform	J Am Med Inform Assoc. 2002 Jul-Aug;9(4):359-68	12087117
2002	Ray HN, Boxwala AA, Anantraman V, Ohno-Machado L	Providing context-sensitive decision-support based on WHO guidelines	Proc AMIA Symp. 2002:637-41	12463901
2002	Das AK, Musen MA	SYNCHRONUS: a reusable software module for temporal integration	Proc AMIA Symp. 2002:195-9	12463814
2003	Poon EG, Wang SJ, Gandhi TK, Bates DW, Kuperman GJ	Design and implementation of a comprehensive outpatient Results Manager	J Biomed Inform. 2003 Feb-Apr;36(1-2):80-91	14552849
2003	Siaw-Teng Liaw, Nabil Sulaiman, Christopher Pearce, Jane Sims, Keith Hill, Heather Grain, Justin Tse, Choon-Kiat Ng	Falls prevention within the Australian general practice data model: methodology, information model, and terminology issues	J Am Med Inform Assoc. Sep-Oct 2003;10(5):425-32	12807809

2003	Barth C, Tobman M, Nätscher C, Sussmann H, Horsch A	Fusing a systematic and a case-based repository for medical decision support	Stud Health Technol Inform. 2003;95:560-4	14664046
2004	Greenes RA, Sordo M, Zaccagnini D, Meyer M, Kuperman GJ	Design of a standards-based external rules engine for decision support in a variety of application contexts: report of a feasibility study at Partners HealthCare System	Stud Health Technol Inform. 2004;107(Pt 1):611-5	15360885
2006	Ebrahiminia V, Riou C, Seroussi B, Bouaud J, Dubois S, Falcoff H, Venot A	Design of a decision support system for chronic diseases coupling generic therapeutic algorithms with guideline-based specific rules	Stud Health Technol Inform. 2006;124:483-8.	17108565
2006	Kashyap V, Morales A, Hongsermeier T	On implementing clinical decision support: achieving scalability and maintainability by combining business rules and ontologies	AMIA Annu Symp Proc. 2006;2006:414-8	17238374
2007	K. Verlaene, W. Joosen and P. Verbaeten	Arriclides: An Architecture Integrating Clinical Decision Support Models	HICSS '07: Proceedings of the 40th Annual Hawaii International Conference on System Sciences January 2007, pp 135	ACM
2007	S. R. Abidi	Ontology-Based Modeling of Breast Cancer Follow-up Clinical Practice Guideline for Providing Clinical Decision Support	CBMS '07: Proceedings of the Twentieth IEEE International Symposium on Computer-Based Medical Systems June 2007, pp 542–547	ACM
2007	P Jannin, X Morandi	Surgical models for computer-assisted neurosurgery	Neuroimage. 2007 Sep 1;37(3):783-91	17613249
2007	Michael Stacey, Carolyn McGregor, Mark Tracy	An architecture for multi-dimensional temporal abstraction and its application to support neonatal intensive care	Annu Int Conf IEEE Eng Med Biol Soc. 2007;2007:3752-6	18002814
2007	Papageorgiou E, Stylios C, Groumpos P	Novel Architecture for supporting medical decision making of different data types based on Fuzzy Cognitive Map Framework	Annu Int Conf IEEE Eng Med Biol Soc. 2007;2007:1192-5	18002176
2008	Chiara Cornalba, Roberto G Bellazzi, Riccardo Bellazzi	Building a normative decision support system for clinical and operational risk management in hemodialysis	IEEE Trans Inf Technol Biomed. 2008 Sep;12(5):678-86	18779083
2009	Farion K, Michalowski W, Wilk S, O'Sullivan D, Rubin S, Weiss D	Clinical decision support system for point of care use--ontology-driven design and software implementation	Methods Inf Med. 2009;48(4):381-90	19448882

2009	Tien Tuan Dao, Frédéric Marin, Marie Christine Ho Ba Tho	Clinical validated Computer-Aided Decision System to the clubfeet deformities	Annu Int Conf IEEE Eng Med Biol Soc. 2009;2009:6230-3	19965086
2009	Q. Zhou	A Clinical Decision Support System for Metabolism Synthesis	CINC '09: Proceedings of the 2009 International Conference on Computational Intelligence and Natural Computing - Volume 02 June 2009, pp 323–325	ACM
2009	Carenini M; ReMINE Consortium	ReMINE: an ontology-based risk management platform	Stud Health Technol Inform. 2009;148:32-42	19745233
2010	Lee J, Kim J, Cho I, Kim Y	Integration of workflow and rule engines for clinical decision support services	Stud Health Technol Inform. 2010;160(Pt 2):811-5	20841798
2010	F. Ongenae, T. Dhaene, F. D. Turck, D. Benoit and J. Decruyenaere	Design of a probabilistic ontology-based clinical decision support system for classifying temporal patterns in the ICU: A sepsis case study	CBMS '10: Proceedings of the 2010 IEEE 23rd International Symposium on Computer-Based Medical Systems October 2010, pp 389–394	ACM
2010	Basilakis J, Lovell NH, Redmond SJ, Celler BG	Design of a decision-support architecture for management of remotely monitored patients	IEEE Trans Inf Technol Biomed. 2010 Sep;14(5):1216-26	20615815
2010	Szymon Wilk, Wojtek Michalowski, Ken Farion, Jelber Sayyad Shirabad	MET3-AE system to support management of pediatric asthma exacerbation in the emergency department	Stud Health Technol Inform. 2010;160(Pt 2):841-5	20841804
2010	Borbolla D, Otero C, Lobach DF, Kawamoto K, Gomez Saldaño AM, Staccia G, Lopez G, Figar S, Luna D, Bernaldo de Quiros FG	Implementation of a clinical decision support system using a service model: results of a feasibility study	Stud Health Technol Inform. 2010;160(Pt 2):816-20	20841799
2011	M.-M. Bouamrane, A. Rector and M. Hurrell	Using OWL ontologies for adaptive patient information modelling and preoperative clinical decision support	Knowledge and Information Systems (KAIS), Volume 29, Issue 2 November 2011, pp 405–418	ACM

2011	Tien Tuan Dao, Frédéric Marin, Henri Bensahel, Marie Christine Ho Ba Tho	Computer-aided decision system for the clubfeet deformities	Adv Exp Med Biol. 2011;696:623-35	21431604
2011	Cao F, Sun X, Wang X, Li B, Li J, Pan Y	Ontology-based knowledge management for personalized adverse drug events detection	Stud Health Technol Inform. 2011;169:699-703	21893837
2011	Chang-Shing Lee, Mei-Hui Wang	A fuzzy expert system for diabetes decision support application	IEEE Trans Syst Man Cybern B Cybern. 2011 Feb;41(1):139-53	20501347
2012	Riaño D, Real F, López-Vallverdú JA, Campana F, Ercolani S, Mecocci P, Annicchiarico R, Caltagirone C	An ontology-based personalization of health-care knowledge to support clinical decisions for chronically ill patients	J Biomed Inform. 2012 Jun;45(3):429-46	22269224
2012	Bright TJ, Yoko Furuya E, Kuperman GJ, Cimino JJ, Bakken S	Development and evaluation of an ontology for guiding appropriate antibiotic prescribing	J Biomed Inform. 2012 Feb;45(1):120-8	22019377
2012	Koutkias V, Kilintzis V, Stalidis G, Lazou K, Niès J, Durand-Texte L, McNair P, Beuscart R, Maglaveras N	Knowledge engineering for adverse drug event prevention: on the design and development of a uniform, contextualized and sustainable knowledge-based framework	J Biomed Inform. 2012 Jun;45(3):495-506	22326287
2012	Grando A, Farrish S, Boyd C, Boxwala A	Ontological approach for safe and effective polypharmacy prescription	AMIA Annu Symp Proc. 2012;2012:291-300. Epub 2012 Nov 3	23304299
2012	Chniti A, Boussadi A, Degoulet P, Albert P, Charlet J	Pharmaceutical validation of medication orders using an OWL Ontology and Business Rules	Stud Health Technol Inform. 2012;180:1224-6	22874408
2012	Paterno MD, Goldberg HS, Simonaitis L, Dixon BE, Wright A, Rocha BH, Ramelson HZ, Middleton B	Using a service oriented architecture approach to clinical decision support: performance results from two CDS Consortium demonstrations	AMIA Annu Symp Proc. 2012;2012:690-8. Epub 2012 Nov 3	23304342
2013	Haug PJ, Ferraro JP, Holmen J, Wu X, Mynam K, Ebert M, Dean N, Jones J	An ontology-driven, diagnostic modeling system	J Am Med Inform Assoc. 2013 Jun;20(e1):e102-10	23523876

2013	W. Yao and A. Kumar	CONFlexFlow: Integrating Flexible clinical pathways into clinical decision support systems using context and rules	Decision Support Systems (DSSY), Volume 55, Issue 2 May, 2013, pp 499–515	ACM
2013	A. Artetxe, E. Sanchez, C. Toro, C. Sanín, E. Szczerbicki, M. Graña and J. Posada	Impact of reflexive ontologies in semantic clinical decision support systems	Cybernetics and Systems (CSYS), Volume 44, Issue 2-3 March 2013, pp 187–203	ACM
2013	Farkash A, Timm JT, Waks Z	A model-driven approach to clinical practice guidelines representation and evaluation using standards	Stud Health Technol Inform. 2013;192:200-4	23920544
2013	Sáez C, Bresó A, Vicente J, Robles M, García-Gómez JM	An HL7-CDA wrapper for facilitating semantic interoperability to rule-based Clinical Decision Support Systems	Comput Methods Programs Biomed. 2013 Mar;109(3):239-49	23199936
2013	Corrigan D, Taweel A, Fahey T, Arvanitis T, Delaney B	An ontological treatment of clinical prediction rules implementing the Alvarado score	Stud Health Technol Inform. 2013;186:103-7	23542977
2013	Reza Shojanoori, Radmila Juric	Semantic remote patient monitoring system	Telemed J E Health. 2013 Feb;19(2):129-36	23363406
2013	S Wilk, W Michalowski, D O'Sullivan, K Farion, J Sayyad-Shirabad, C Kuziemsky, B Kukawka	A task-based support architecture for developing point-of-care clinical decision support systems for the emergency department	Methods Inf Med. 2013;52(1):18-32	23232759
2013	Özgün Yılmaz, Rıza Cenk Erdur, Mustafa Türksever	SAMS--a systems architecture for developing intelligent health information systems	J Med Syst. 2013 Dec;37(6):9989	24197356
2014	Bau CT, Chen RC, Huang CY	Construction of a clinical decision support system for undergoing surgery based on domain ontology and rules reasoning	Telemed J E Health. 2014 May;20(5):460-72	24730353
2014	Hua-qiong Wang, Tian-shu Zhou, Li-li Tian, Yang-ming Qian, Jing-song Li	Creating hospital-specific customized clinical pathways by applying semantic reasoning to clinical data	J Biomed Inform. 2014 Dec;52:354-63	25109270
2014	Sesen MB, Peake MD, Banares-Alcantara R, Tse D, Kadir T, Stanley R, Gleeson F, Brady M	Lung Cancer Assistant: a hybrid clinical decision support application for lung cancer care	J R Soc Interface. 2014 Sep 6;11(98):20140534	24990290
2014	M. Gallerani, D. Pelizzola, M. Pivanti, M.	Appropriateness of Repeated Execution of Laboratory Examinations: A CDSS Approach	ICHI '14: Proceedings of the 2014 IEEE International Conference on Healthcare	ACM

	Boni, E. Lamma and E. Bellodi		Informatics September 2014, pp 161–169	
2014	Goldberg HS, Paterno MD, Rocha BH, Schaeffer M, Wright A, Erickson JL, Middleton B	A highly scalable, interoperable clinical decision support service	J Am Med Inform Assoc. 2014 Feb;21(e1):e55-62	23828174
2014	Samuel Alan Stewart, Samina Abidi, Louise Parker, Mark Bernstein, Syed Sibte Raza Abidi	Clinical guideline-driven personalized self-management diary for paediatric cancer survivors	Stud Health Technol Inform. 2014;205:18-22	25160137
2015	Ying Shen, Joël Colloc, Armelle Jacquet-Andrieu, Kai Lei	Emerging medical informatics with case-based reasoning for aiding clinical decision in multi-agent system	J Biomed Inform. 2015 Aug;56:307-17	26133480
2015	Brendan C Delaney, Vasa Curcin, Anna Andreasson, Theodoros N Arvanitis, Hilde Bastiaens, Derek Corrigan, Jean-Francois Ethier, Olga Kostopoulou, Wolfgang Kuchinke, Mark McGilchrist, Paul van Royen, Peter Wagner	Translational Medicine and Patient Safety in Europe: TRANSFoRm--Architecture for the Learning Health System in Europe	Biomed Res Int. 2015;2015:961526	26539547
2015	Borna Jafarpour, Samina Raza Abidi, Ahmad Marwan Ahmad, Syed Sibte Raza Abidi	INITIATE: An Intelligent Adaptive Alert Environment	Stud Health Technol Inform. 2015;216:285-9	26262056
2015	Vladimir Robles-Bykbaev, Martín López-Nores, José Pazos-Arias, Diego Quisi-Peralta, Jorge García-Duque	An Ecosystem of Intelligent ICT Tools for Speech-Language Therapy Based on a Formal Knowledge Model	Stud Health Technol Inform. 2015;216:50-4	26262008
2016	Szymon Wilk, Mounira Kezadri-Hamiaz, Daniela Rosu, Craig Kuziemsky, Wojtek Michalowski,	Using Semantic Components to Represent Dynamics of an Interdisciplinary Healthcare Team in a Multi-Agent Decision Support System	J Med Syst. 2016 Feb;40(2):42	26590980

	Daniel Amyot, Marc Carrier			
2016	Samina Raza Abidi, Jafna Cox, Ashraf Abusharekh, Nima Hashemian, Syed Sibte Raza Abidi	A Digital Health System to Assist Family Physicians to Safely Prescribe NOAC Medications	Stud Health Technol Inform. 2016;228:519-23	27577437
2016	Zhang YF, Tian Y, Zhou TS, Araki K, Li JS	Integrating HL7 RIM and ontology for unified knowledge and data representation in clinical decision support systems	Comput Methods Programs Biomed. 2016 Jan;123:94-108	26474836
2016	Goldberg HS, Paterno MD, Grundmeier RW, Rocha BH, Hoffman JM, Tham E, Swietlik M, Schaeffer MH, Pabbathi D, Deakyne SJ, Kuppermann N, Dayan PS	Use of a remote clinical decision support service for a multicenter trial to implement prediction rules for children with minor blunt head trauma	Int J Med Inform. 2016 Mar;87:101-10	26806717
2016	Luis Marco-Ruiz, Carlos Pedrinaci, J A Maldonado, Luca Panziera, Rong Chen, J Gustav Bellika	Publication, discovery and interoperability of Clinical Decision Support Systems: A Linked Data approach	J Biomed Inform. 2016 Aug;62:243-64	27401856
2017	Zhang YF, Gou L, Zhou TS, Lin DN, Zheng J, Li Y, Li JS	An ontology-based approach to patient follow-up assessment for continuous and personalized chronic disease management	J Biomed Inform. 2017 Aug;72:45-59	28676255
2017	Shang Y, Wang Y, Gou L, Wu C, Zhou T, Li JS	Development of a Service-Oriented Sharable Clinical Decision Support System Based on Ontology for Chronic Disease	Stud Health Technol Inform. 2017;245:1153-1157	29295283
2017	Chen RC, Jiang HQ, Huang CY, Bau CT	Clinical Decision Support System for Diabetes Based on Ontology Reasoning and TOPSIS Analysis	J Healthc Eng. 2017;2017:4307508	29312655
2017	Kopanitsa G	Integration of Hospital Information and Clinical Decision Support Systems to Enable the Reuse of Electronic Health Record Data	Methods Inf Med. 2017 May 18;56(3):238-247	28361157
2017	Samina Abidi	A Knowledge-Modeling Approach to Integrate Multiple Clinical Practice Guidelines to Provide Evidence-Based Clinical Decision Support for Managing Comorbid Conditions	J Med Syst. 2017 Oct 26;41(12):193	29076113
2018	Shen Y, Yuan K, Chen D, Colloc J, Yang M, Li Y, Lei K	An ontology-driven clinical decision support system (IDDAP) for infectious disease diagnosis and antibiotic prescription	Artif Intell Med. 2018 Mar;86:20-32	29433958

2018	Hirenkumar Nakawala, Giancarlo Ferrigno, Elena De Momi	Development of an intelligent surgical training system for Thoracentesis	Artif Intell Med. 2018 Jan;84:50-63	29169646
2018	Brigitte Séroussi, Gilles Guézennec, Jean-Baptiste Lamy, Naiara Muro, Nekane Larburu, Booma Devi Sekar, Coralie Prebet, Jacques Bouaud	Reconciliation of multiple guidelines for decision support: a case study on the multidisciplinary management of breast cancer within the DESIREE project	AMIA Annu Symp Proc. 2018 Apr 16;2017:1527-1536	29854222
2018	Winter A, Stäubert S, Ammon D, Aiche S, Beyan O, Bischoff V, Daumke P, Decker S, Funkat G, Gewehr JE, de Greiff A, Haferkamp S, Hahn U, Henkel A, Kirsten T, Klöss T, Lippert J, Löbe M, Lowitsch V, Maassen O, Maschmann J, Meister S, Mikolajczyk R, Nüchter M, Pletz MW, Rahm E, Riedel M, Saleh K, Schuppert A, Smers S, Stollenwerk A, Uhlig S, Wendt T, Zenker S, Fleig W, Marx G, Scherag A, Löffler M	Smart Medical Information Technology for Healthcare (SMITH)	Methods Inf Med. 2018 Jul;57(S 01):e92-e105	30016815
2018	Brigitte Seroussi, Jean-Baptiste Lamy, Naiara Muro, Nekane Larburu, Booma Devi Sekar, Gilles Guézennec, Jacques Bouaud	Implementing Guideline-Based, Experience-Based, and Case-Based Approaches to Enrich Decision Support for the Management of Breast Cancer Patients in the DESIREE Project	Stud Health Technol Inform. 2018;255:190-194	30306934
2018	Wenquan Jin, Do Hyeun Kim	Design and Implementation of e-Health System Based on Semantic Sensor Network Using IETF YANG	Sensors (Basel). 2018 Feb 20;18(2):629	29461493

2019	Román-Villarán E, Pérez-Leon FP, Escobar-Rodriguez GA, Martínez-García A, Álvarez-Romero C, Parra-Calderón CL	An Ontology-Based Personalized Decision Support System for Use in the Complex Chronically Ill Patient	Stud Health Technol Inform. 2019 Aug 21;264:758-762	31438026
2019	Semenov I, Osenev R, Gerasimov S, Kopanitsa G, Denisov D, Andreychuk Y	Experience in Developing an FHIR Medical Data Management Platform to Provide Clinical Decision Support	Int J Environ Res Public Health. 2019 Dec 20;17(1):73	31861851
2019	Nguyen BP, Reese T, Decker S, Malone D, Boyce RD, Beyan O	Implementation of Clinical Decision Support Services to Detect Potential Drug-Drug Interaction Using Clinical Quality Language	Stud Health Technol Inform. 2019 Aug 21;264:724-728	31438019
2019	Borna Jafarpour, Samina Raza Abidi, William Van Woensel, Syed Sibte Raza Abidi	Execution-time integration of clinical practice guidelines to provide decision support for comorbid conditions	Artif Intell Med. 2019 Mar;94:117-137	30871678
2019	El-Sappagh S, Ali F, Hendawi A, Jang JH, Kwak KS	A mobile health monitoring-and-treatment system based on integration of the SSN sensor ontology and the HL7 FHIR standard	BMC Med Inform Decis Mak. 2019 May 10;19(1):97	31077222
2020	Maldonado JA, Marcos M, Fernández-Breis JT, Giménez-Solano VM, Legaz-García MDC, Martínez-Salvador B.	CLIN-IK-LINKS: A platform for the design and execution of clinical data transformation and reasoning workflows.	Comput Methods Programs Biomed. 2020;**197**:105616	32629294
