# Supplementary figures and images for: Ontologies Applied in Clinical Decision Support System Rules: Systematic Review
Source: JMIR Med Inform. 2023 Jan 19;11:e43053. doi: 10.2196/43053 (PMC9896360; doi:10.2196/43053)

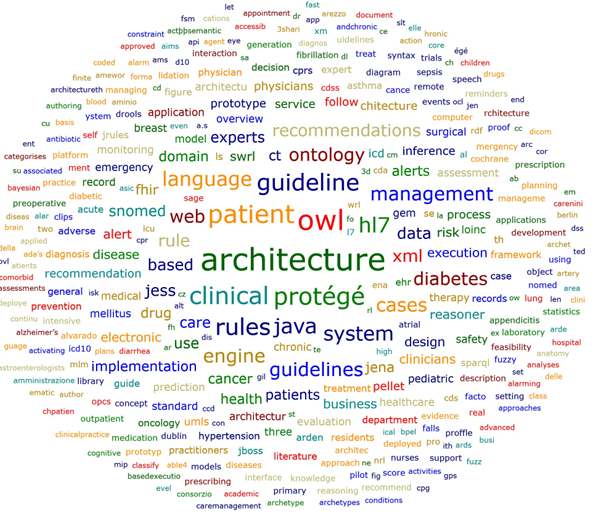

Supplement: Multimedia Appendix 6 [file medinform_v11i1e43053_app6.png]

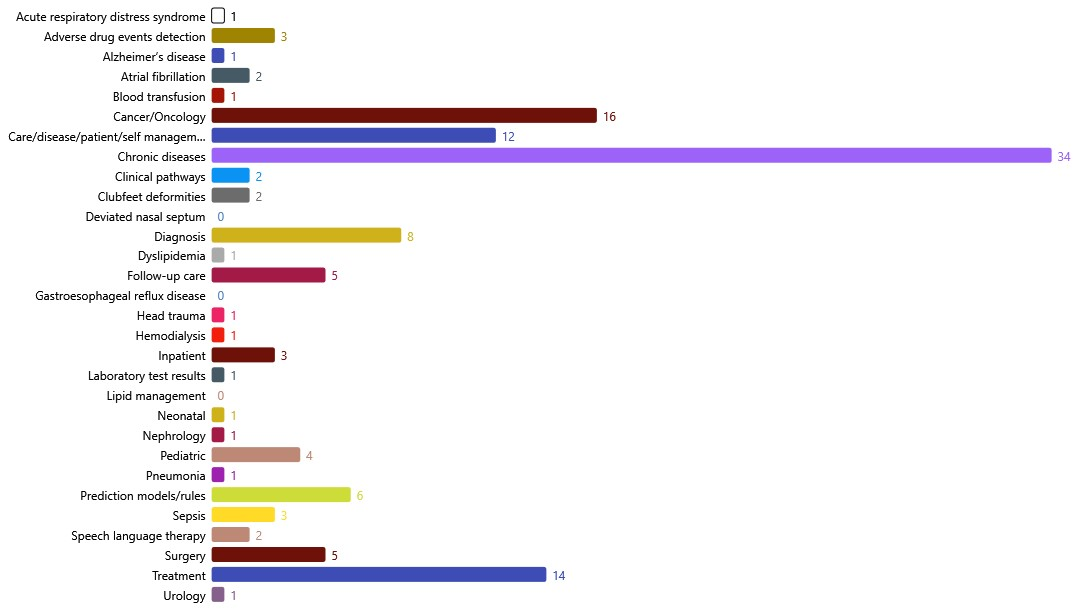

Supplement: Multimedia Appendix 7 [file medinform_v11i1e43053_app7.png]
