# Supplementary material for: Ontologies Applied in Clinical Decision Support System Rules: Systematic Review
Source: JMIR Med Inform. 2023 Jan 19;11:e43053. doi: 10.2196/43053 (PMC9896360; doi:10.2196/43053)
Supplement: Multimedia Appendix 8 [file medinform_v11i1e43053_app8.pdf]

Appendix 8 Basic CDSS profiles in included publications (*n* = 81)

**Authors**	**Year**	**CDSS is used for (Domain)**	**CDSS is used as**	**CDSS is used by**	**Architecture design (production/prototype)***	**CDSS evaluation**
Abidi SS, Manickam S [52]	2000	The automatic transformation from XML to clinical cases	Transformation tool	-	Architecture and implementation	-
De Clercq PA, Blom JA, Hasman A, et al [53]	2000	Drug interactions or drug contraindication in ICU, family medicine, and psychiatry	Alerts	Physicians	Production	A hospital in the Netherlands
Payne TH, Savarino J, Marshall R, et al. [54]	2000	Medication errors prevention and detection	Alerts, reminders, notifications to clinicians	Practitioners	In production, Veterans Affairs hospital	-
Riesco AM, Tomás RM, Mira MJ [51]	2000	Therapeutic decision analysis in oncology	Recommendation of ideal therapy	Physicians	-	-
Achour SL, Dojat M, Rieux C, et al [55]	2001	Safe blood transfusion in hospital settings	Alerts, consultation, recommendations	Physicians	Prototype	30 orders were initially studied; 20 orders were after modifications
Séroussi B, Bouaud J, Dréau H, et al [56]	2001	Prevention of medication order error in primary care	Recommendations	General practitioners	Prototype under development	A simulated case

Karadimas HC, Chailloleau C, Hemery F, et al [57]	2002	Urology and nephrology as examples	Urgent or serious alerts	Physicians	Integrated into CIS and patient record systems	Tested in urology and nephrology departments and stand-alone used for education.
Ray HN, Boxwala AA, Anantraman V, et al [31]	2002	Diarrhea management	Patient assessment, treatment plan recommendations	Clinicians	CDSS integrated with a mini EMR	Plan
Das AK, Musen MA [58]	2002	Integration of time-related queries for clinical data	Integrated temporal query for clinical decision support	-	Software module	-
Barth C, Tobman M, Nätscher C, et al [32]	2003	Right diagnosis or treatment	Recommendations	Gastroenterologists	Prototype, web-accessible	Patient cases
Liaw ST, Sulaiman N, Pearce C, et al [60]	2003	Falls prevention	Recommend care plans	-	Prototype, Web API	Testing by clinicians and patients, and groups
Poon EG, Wang SJ, Gandhi TK, et al [59]	2003	To review and act on laboratory test results.	Manage abnormal results, generate letters to patients, send reminders for future tests	Outpatient physicians	Production	Pilot studies in two clinics with 20 physicians and nurses
Greenes RA, Sordo M, Zaccagnini D, et al [61]	2004	Centralized rule base and rule engine for CDS, e.g., medications, lab	Alerts, reminders	Physicians	Prototype	Case analysis

Ebrahiminia V, Riou C, Seroussi B, et al [62]	2006	Therapeutic plan for type 2 diabetes and hypertension	Recommendations for prescriptions	Clinicians	-	-
Kashyap V, Morales A, Hongsermeier T [63]	2006	Lipid management	Clinical recommendation or order	Clinicians	Production	Ontology inference performance
Abidi SR [65]	2007	Breast cancer follow-up care by PCP	Patient-specific recommendations	PCP	Production, two PCP clinics	-
Jannin P, Morandi X [66]	2007	Prediction of surgical positions in neurosurgery	Neurosurgeons	Neurosurgeons	Prototype	159 surgical cases
Papageorgiou E, Stylios C, Groumpos P [68]	2007	Generic medical decision making support	Recommendations	Clinicians	Design	-
Stacey M, McGregor C, Tracy M [67]	2007	Neonatal intensive care	Early detection of sepsis, pneumothorax, and periventricular leukomalacia alerts	Clinicians	Prototype	Hypothetical case and hospital testing
Verlaene K, Joosen W, Verbaeten P [64]	2007	Generic design, two use cases: medication order and clinical pathway	Recommendations/suggestions	Clinicians	Prototype	Two scenarios
Cornalba C, Bellazzi RG, Bellazzi R [69]	2008	Risk management in hemodialysis	Suggestions for the risk profile of a patient and optimal decision	Clinicians, nephrologists	Design	Examples and case studies
Carenini M [73]	2009	Prediction, detection, monitoring of	Alerts and notifications on efficient reaction or response to a risk situation	Clinicians	Ongoing design and ontology building	-

		patient risk management				
Dao TT, Marin F, Ho Ba Tho MC [71]	2009	Assessment, evaluation, treatment of clubfeet	Diagnosis, conservative treatment, and follow up	Patients and experts	Web-based system	Over 1000 cases used
Farion K, Michalowski W, Wilk S, et al [70]	2009	Acute pain in the emergency room, postoperative management of prostatectomy	Triage recommendations	Clinicians	Prototype	Feasibility
Zhou Q [72]	2009	Metabolism synthesis, diabetes, hypertension, hyperlipidemia, obesity, hyperuricemia	Recommendations	Clinicians	Experimental system	20 clinical cases + 20 physicians
Basilakis J, Lovell NH, Redmond SJ, et al. [76]	2010	Telehealth, patient monitoring, health risk stratification to better manage the patient with chronic conditions	Alert, report, workflow modification	Clinicians	Framework, prototype	One case study
Borbolla D, Otero C, Lobach DF, et al [26]	2010	Preventive services, breast cancer screening	Recommendations, reminders	Physicians	Feasibility of Web service for SEBASTIAN incorporated with EMR in a hospital in Italy, production	Manually reviewed 210 patient cases

Lee J, Kim J, Cho I, et al [74]	2010	General design with a specific example in a laboratory context and hypertension	Alerts, recommendations	Clinicians	Prototype, testbed	323445 test cases
Ongenae F, Dhaene T, De Turck F, et al [75]	2010	ICU, sepsis, pattern detection in time series	Suggestions on time-related trends or notification of pathology classification	ICU clinicians	Framework	ICU use case
Wilk S, Michalowski W, Farion K, et al [77]	2010	Management of pediatric asthma exacerbation in the emergency department	Collect data, evaluate exacerbation severity, plan treatment	Emergency physicians	Simulated setting in hospital with HIS	Testing, 120 visits in 12 hours
Bouamrane MM, Rector A, Hurrell M [78]	2011	Preoperative risk assessment	Risk assessment, clinical recommendations	Clinicians	Software, mentioned implementation in practice	Example patient
Cao F, Sun X, Wang X, et al [80]	2011	Detect adverse drug events	ADE search and report	Clinicians	Production, web-based applications	-
Dao TT, Marin F, Bensahel H, et al [79]	2011	Assessment, evaluation, treatment of clubfeet, pediatric orthopedics	To generate a diagnosis, conservative treatment	Clinicians	Web application	Over 1000 cases
Lee CS, Wang MH [81]	2011	Diabetes diagnosis	Semantic description	-	Developed expert system, prototype	Experiments with cases
Bright TJ, Yoko Furuya E, Kuperman GJ, et al [83]	2012	Prescribing antibiotics	Alerts	Clinicians	Prototype	81 patient records

Chniti A, Boussadi A, Degoulet P, et al [86]	2012	Medication orders	Suggestions on whether a prescription is valid	Clinicians	Prototype in hospital	Use case
Grando A, Farrish S, Boyd C, et al. [85]	2012	ADE reduction; safe and effective multi-drug treatment, COPD, type 2 diabetes, osteoporosis, hypertension	Recommendations	Clinicians with prescription rights	Prototype	Via evaluation scenarios
Koutkias V, Kilintzis V, Stalidis G, et al [84]	2012	Prevent adverse drug events	Alerts and recommendations	Clinical personnel	Knowledge framework	By experts and test cases; knowledge base authors testing the results via studies
Paterno MD, Goldberg HS, Simonaitis L, et al [87]	2012	Chronic condition management, diabetes, or coronary artery disease	Alerts, reminders	Clinicians	Production	Partners HealthCare, Wishard Memorial Hospital
Riaño D, Real F, López-Vallverdú JA, et al [82]	2012	Chronic comorbidities management	Personalized care plan recommendations	Healthcare professionals	Production systems in Italy, part of the K4CARE project	916 patients were used to evaluate the CDSS
Artetxe A, Sanchez E, Toro C, et al [89]	2013	Diagnosis of Alzheimer's disease	-	Physicians	Production, Spanish research project	Ten patients

Corrigan D, Taweel A, Fahey T, et al. [92]	2013	Clincial prediction rules, Alvarado score	Clinical prediction	Clinicians	-	Three use cases
Farkash A, Timm JT, Waks Z [90]	2013	Hypertension	Recommendations, suggestions	Clinicians	Prototype	No user interface yet, two use cases for future
Haug PJ, Ferraro JP, Holmen J, et al [33]	2013	Pneumonia	Recommendation of diagnosis	Clinicians	An experimental system	Tested by sample patient data
Sáez C, Bresó A, Vicente J, et al. [91]	2013	Manage outpatient diabetes care in a telemedicine system	Recommendations for patient lifestyles on risks of diabetes	Physicians, patients	Production- telemedicine platform in Spanish	Ten patient records
Shojanoori R, Juric R [93]	2013	Remote patient monitoring system at care homes	Alarms, reminders, recommendations, or activating devices (e.g., heater)	Healthcare professionals	Experiment system	Preventive e-healthcare example
Wilk S, Michalowski W, O'Sullivan D, et al. [94]	2013	Triage, diagnosis, therapeutic plan, disposition in the emergency room; management of pediatric asthma exacerbations	Recommended actions, suggestions, patient data management in the emergency department	Emergency department physicians	Pilot in production	102 patients enrolled
Yao W, Kumar A [88]	2013	Generic clinical workflow, heart failure, diagnosis, and treatment	Recommendations	Healthcare professionals	Prototype	Example of patients

Yılmaz Ö, Erdur RC, Türksever M [95]	2013	Assistance in diagnosis and treatment	Recommendations	Physicians, dentists	Developed system based on SAMS	A dental scenario
Bau CT, Chen RC, Huang CY [96]	2014	Management of diabetes inpatients during surgery	Recommendations	Healthcare professionals	Prototype	12 clinicians and 20 de-identified patients
Gallerani M, Pelizzola D, Pivanti M, et al [99]	2014	Laboratory repeated examination	Recommendation, warning on the appropriateness of the laboratory test	Physicians	Embedded in computerized physician order entry in two Italian hospitals, production	Three months of testing in two hospitals
Goldberg HS, Paterno MD, Rocha BH, et al [27]	2014	Chronic condition management, immunization schedules, pediatric head trauma	Recommendations, suggestions	Clinicians or patients	Production	Deployed at Partners HealthCare and trialed in multiple sites
Sesen MB, Peake MD, Banares-Alcantara R, et al [98]	2014	Lung cancer care, treatment selection	Recommendations	Physicians/experts	Prototype with a web interface	4020 patient records retrospectively
Stewart SA, Abidi S, Parker L, et al [100]	2014	Pediatric cancer follow-up management	Recommendations	Patients	Embedded in an EHR portal in Canada	Evaluated by nurses and oncologists on content; domain experts evaluated rules
Wang HQ, Zhou TS, Tian LL, et al [97]	2014	A clinical pathway for deviated nasal septum	Provide more efficient and intelligent clinical procedures	Healthcare professionals	-	224 patients with 11473 orders were selected to use/test

Delaney BC, Curcin V, Andreasson A, et al [102]	2015	Three use cases include diabetes, reflux symptoms, diagnostic support on abdominal pain, chest pain, and shortness of breath	Identifying patients with diabetes providing diagnostic support on effective medication for reflux symptoms.	Healthcare professional	Under development, the TRANSFoRm project	Three use cases and tests in a simulated environment
Jafarpour B, Abidi SR, Ahmad AM, et al [103]	2015	Alert environment avoid alert fatigue	Alerts	Clinicians	Framework	Five general practitioners
Robles-Bykbaeva V, López-Noresb M, Pazos-Ariasb J, et al [104]	2015	Speech-language therapy	Recommendations on therapy plans, generate content for students, monitor and evaluate patient progress	Speech and language pathologists, doctors, students, patients, relatives	In production, five institutions in Ecuador	32 children assessed; 6 experts + 53 children evaluated
Shen Y, Colloc J, Jacquet-Andrieu A, et al [101]	2015	Optimal diagnosis, prognosis, treatment of gastric cancer	Suggestions on diagnosis, prognosis, treatment of gastric cancer	Clinicians and patients	-	Domain experts to provide feedback on CDSS; using 17 gastric cancer cases
Abidi SR, Cox J, Abusharekh A, et al [106]	2016	Safe prescription for patients with atrial fibrillation	Prescription eligibility check and authorization (Canadian), recommendations	Family physicians	Embedded within the IMPACT-AF ecosystem with a web-based stand-alone system	100 de-identified patient cases
Goldberg HS, Paterno MD, Grundmeier RW, et al [108]	2016	Children with minor blunt head trauma	Recommendations	Physicians in the emergency department	One web accessed CDS service integrated with EHR in two sites, production	A multicenter trial

Marco-Ruiz L, Pedrinaci C, Maldonado JA, et al [109]	2016	Methods and models to enhance CDS service-oriented architecture as Linked Services	Alerts, recommendations	Clinicians	Models are complaint to Linked Data principles, semantic representation for CDSS services	The query of CDSS services
Wilk S, Kezadri-Hamiaz M, Rosu D, et al [105]	2016	Management of advanced chronic kidney disease	Identify correct physicians, handle exceptions, execute tasks, and change physicians.	Healthcare professionals, nephrologists, cardiologists, dieticians, nurse	Proof of concept implementation in OpenMRS	Simulated scenarios, fictitious patient data
Zhang YF, Tian Y, Zhou TS, et al [107]	2016	Inpatient management of type 2 diabetes	Recommendations for care plan	Clinicians	Prototype	100 patient cases
Abidi S [114]	2017	Clinical management of comorbidities, including atrial fibrillation and chronic heart failure	Recommendations for safe and effective plans for comorbidity therapeutics	Family physicians	Web-based CDSS; stand-alone system	Domain experts on content; usability of CDSS by 10 PCP
Chen RC, Jiang HQ, Huang CY, et al [112]	2017	Diabetes management, outpatient	Recommendations on HbA1c target and medications	Endocrinologists or other physicians	Web system as a prototype	Ten patient records
Kopanitsa G [113]	2017	Treatment quality control as a use case	Check accuracy of mapping, transforming, and integrating data from multiple EHR	Clinicians	Production	3436 treatment case records evaluated
Shang Y, Wang Y, Gou L, et al [111]	2017	Type 2 diabetes and hypertension in patient management	Suggestions for treatment decisions or lifestyles	Clinicians	Production, web services	Use cases
Zhang YF, Gou L, Zhou TS, et al [110]	2017	Chronic disease management,	Recommended or suggested assessment/plan	Clinicians and patients	Deployed system, web service	Case study of patients with type 2 diabetes

		follow-up assessment				(36162), follow-up assessments
Jin W, Kim DH [120]	2018	e-health services, sensors, device interoperability	Messaging	Healthcare professionals or patients	Prototype	Tested
Nakawala H, Ferrigno G, De Momi E [116]	2018	Surgical training system for thoracentesis	Surgical procedures planning, execution, practicing	Surgical trainees	Prototype	10 participants in two experiments
Séroussi B, Guézennec G, Lamy JB, et al [117]	2018	Breast cancer management	Recommendations, action plans	Clinicians	Web API, in production	Case studies
Séroussi B, Lamy JB, Muro N, et al [119]	2018	Breast cancer management	Recommend best care plan for breast cancer patients	Clinicians	Web API, in production	Use cases
Shen Y, Yuan K, Chen D, et al [115]	2018	Infectious disease diagnosis, antibiotic prescription	Suggestions	Patients	-	Compare against other ontologies: DO, IDO, ROC for 215 infectious diseases; 84 records
Winter A, Staubert S, Ammon D, et al [118]	2018	Acute respiratory distress syndrome (ARDS)	Suggestions on mechanical ventilation; surveillance of ICU patients	ICU clinicians	Architecture framework	Use case for analysis
El-Sappagh S, Ali F, Hendawi A, et al [124]	2019	Monitor and manage type 1 diabetes	Real-time patient monitoring and care plan and lifestyle recommendation	Clinicians and patients	Cloud-based EHR system	Scenario

Jafarpour B, Raza Abidi S, Van Woensel W, et al [123]	2019	Safe and efficient comorbidity management	Recommendations	Clinicians	-	Local CIG integration engine test + surveys
Nguyen BP, Reese T, Decker S, et al [29]	2019	Detect potential drug-drug interactions	-	Clinicians	-	Two implementation use cases
Román-Villarán E, Pérez-Leon FP, Escobar-Rodriguez GA, et al [121]	2019	Manage cases of complex chronic illness	Recommendation and personalized treatment plans	Clinicians	Part of the PITeS-TIiSS project, in the production system in Spain, DIRAYA	-
Semenov I, Osenev R, Gerasimov S, et al [122]	2019	Improve efficiency and efficacy of health care delivery	Recommendations, suggestions	Clinicians and patients	Production system connected with HIS	15000 orders per day
Maldonado JA, Marcos M, Fernández-Breis JT, et al [125]	2020	Normalize clinical lab results; risk assessment for colorectal cancer screening	Support clinical data transformation to different formats;	Clinicians	Web-based platform	Two use cases with actual patients data

* Information more granular than production systems in this column implies the production systems.

Abbreviations: -, not specified; API: application programming interface; CDSS: clinical decision support systems; CIS: clinical information systems; COPD: chronic obstructive pulmonary disease; CPOE: computerized physician order entry; DIRAYA: the Electronic Health Record system within the Andalusian Public Healthcare System; DO: disease ontology; EMR: electronic medical records; EHR: electronic health records; ER: emergency rooms; HIS: hospital information system; HPO: human phenotype ontology; ICU: intensive care units; IDO: infectious disease ontology; KB: knowledge base; PCP: primary care providers; ROC: receiver operating characteristics; VA: Veterans Affairs.
