# Supplementary material for: Ontologies Applied in Clinical Decision Support System Rules: Systematic Review
Source: JMIR Med Inform. 2023 Jan 19;11:e43053. doi: 10.2196/43053 (PMC9896360; doi:10.2196/43053)
Supplement: Multimedia Appendix 9 [file medinform_v11i1e43053_app9.pdf]

Appendix 9 Comparison of CDSS rule characteristics in included publications (*n* = 54)

**Authors/ year**	**CDSS rule language**	**CDSS rule authoring/ editing**	**Rule sources**	**Rule engine**	**Rule format/ management**	**Interoperability**	**Operation**
De Clercq PA, Blom JA, Hasman A, et al, 2000 [53]	-	Knowledge Base Editor (KBE)	Guidelines	-	-	-	Patient data + events → guidelines ontology, models → recommended actions
Payne TH, Savarino J, Marshall R, et al., 2000 [54]	XML	-	-	-	Microsoft's Component Object Model (COM)	HL7	Medication order + patient data → medication order monitor check → notifications
Achour SL, Dojat M, Rieux C, 2001 [55]	MLM	Newly presented tool	UMLS + experts	Inference engine	Arden	-	Domain ontology → knowledge base → MLM + blood data + patient states
Karadimas HC, Chailloleau C, Hemery F, et al, 2002 [57]	MLM	The Arden/J framework developed by the authors	-	-	Arden	-	-
Ray HN, Boxwala AA, Anantraman V, et al, 2002 [31]	C	-	Guidelines from WHO	Guideline engine	CLIPS	-	Clinician enter data + EMR data + CLIPS rule → guideline engine + site configuration → recommendations
Barth C, Tobman M, Nätscher C, et al, 2003 [32]	Java	-	Textbook, multimedia	Inference engine	-	-	Patient data → inference engine + rules → case-based data + systematic data → recommendations

Liaw ST, Sulaiman N, Pearce C, et al, 2003 [60]	MLM, XML	-	Literature, physicians, and patients' input	-	Arden	HL7, XML	-
Kashyap V, Morales A, Hongsermeier T, 2006 [63]	OWL	-	Guidelines	ILOG, Cerebron (OWL ontology engine)	-	-	Patient data + event → rule engine + ontology engine → recommendations
Abidi SR, 2007 [65]	OWL	Rule authoring model via an interface, self-developed	Guidelines	GEM execution engine for CPG	The rule is specified in the logic element (class) of the CPG ontology	-	Computerize guideline → ontology → A family physician enters a patient's data via the CDSS interface, receives recommendations, explanation
Stacey M, McGregor C, Tracy M, 2007 [67]	XML	Jess	Data mining results and clinicians' input	-	-	-	Patient data + rules + ontology → alerts + archive
Verlaene K, Joosen W, Verbaeten P, 2007 [64]	XML	Eclipse Rich Client Platform, Graphical Editing Framework (GEF), ILOG JRules	Guidelines	Drools v2.4, Java and ILOG JRules v4.6	-	HL7 RIM	Patient data + event + process → rule engine → recommendation
Cornalba C, Bellazzi RG, Bellazzi R, 2008 [69]	-	-	Guidelines, publications	Bayes network	Bayes rules	-	Patient data + monitoring → risk profile → Bayes network → optimal decisions

Carenini M, 2009 [73]	XML	Semantic Business Process Composer (SBPC)	Guidelines and data mining results	Business Process Execution Language (BPEL)	-	HL7	-
Zhu Q, 2009 [72]	XML/SAGE	-	Guidelines	Re-inference	CLIPS rule set	-	Patient data + model → rules + inference → recommendations
Basilakis J, Lovell NH, Redmond SJ, et al., 2010 [76]	-	JBoss AS framework	-	JBoss Rules	jBPM (business process management)	HL7	Clinical measures → rule engine + statistical analysis tool → recommendations
Borbolla D, Otero C, Lobach DF, et al, 2010 [26]	XML	-	Guidelines	-	SEBASTIAN rules	HL7	Patient data + preventive service rules → reminder + explanation
Lee J, Kim J, Cho I, et al, 2010 [74]	SAGE	uEngine, BRAIN	Guidelines	uEngine, BRAIN	-	-	Patient data + events + rule engine + workflow engine → alert or recommendation
Ongenae F, Dhaene T, De Turck F, et al, 2010 [75]	SWRL	-	Galen ontology, medical databases	-	-	-	Patient data over time + ML + semantic reasoning → suggestions
Wilk S, Michalowski W, Farion K, et al, 2010 [77]	-	JADE (Java Agent DEvelopment Framework)	Literature, Cochrane library	-	-	Mirth Connect to enable communication between the MET3-AE and HIS; HL7	-
Bouamrane MM, Rector	OWL	Protégé-OWL	Guidelines	JBoss Rules	-	-	Patient data + CDSS + ontology → risk

A, Hurrell M, 2011 [78]							assessment → recommendations
Cao F, Sun X, Wang X, et al, 2011 [80]	SPARQL queries, RDF	ADE ontology and knowledge base were used J2EE, DB2 V9, WebSphere V7.	Local hospital data, FDA documents, Structured Product Labeling (SPL), Linked Open Drug Data (LODD)	T-Box reasoner; A-Box reasoner	-	-	ADE knowledge + patient data → personalized ADE detection via semantic query, reasoning
Bright TJ, Yoko Furuya E, Kuperman GJ, et al, 2012 [83]	SWRL, queried by SQWRL	Protégé SWRL tab	Recommendation, assertive relational knowledge	Jess Rule Engine	Implemented in Java	-	Prescribing rules + ontology-driven alert module + patient database
Chniti A, Boussadi A, Degoulet P, et al, 2012 [86]	OWL	JRules, execute and manage business rules, JRules OWL plug-in	Business object model (BOM), the executable object model (XOM)	Jena	Java framework	-	OWL ontology/business object model entities + Jena → executable object model
Grando A, Farrish S, Boyd C, et al, 2012 [85]	SWRL, OWL	Protégé	Guidelines on COPD, diabetes mellitus, osteoporosis, and hypertension	Jess reasoner	Implemented in Java	Setting-independent rules	A repository of rules + polypharmacy treatment + ontology + patient data
Koutkias V, Kilintzis V, Stalidis G, et al, 2012 [84]	XML	A frame-based version of Protégé	Clinical guidelines and protocols	Inference engine: including CIG execution engine; Finite State Machine (FSM)-based	-	-	-

				execution engine			
Artetxe A, Sanchez E, Toro C, et al, 2013 [89]	OWL-DL	-	Clinicians	-	-	-	Patient data + lab results → agent matchmaker → diagnosis assistance
Farkash A, Timm JT, Waks Z, 2013 [90]	The Natural Rule Language (NRL)	-	Clinical practice guidelines	-	-	HL7 CCD	Patient data in CDA → service, CDS rules, criteria → treatment recommendations
Sáez C, Bresó A, Vicente J, et al, 2013 [91]	Jess	HL7-CDA wrapper, developed by authors	Rules for physician assessment, rules for patient assessment, intermediate calculus rules, ADA recommendation rules, rule context information	Inference engine, pattern matching		HL7 – CDA input and output	CDA input → binding → inference → binding from rule results to CDA output → CDA output
Shojanoori R, Juric R, 2013 [93]	OWL/SWRL	-	Presented as an example, not in scale design	-	-	-	Patient data + monitoring data + contexts → ontology + reasoning → recommendations
Wilk S, Michalowski W, O'Sullivan D, et al, 2013 [94]	-	-	Requirements by emergency department physicians, guidelines	-	-	HL7 with HIS	Collect data (observation) → reasoning (decision) → action (recommendation)
Yao W, Kumar A, 2013 [88]	SWRL	Protégé + SWRLJessTab	Patient context, clinician context, resource context, location context,	Rule engine-Jess; Workflow	Implemented in Java	-	Data from HIS + medical and context ontologies → rule +

			patient evaluation rule, patient diagnosis rule, patient treatment rule, prescription checking rule	engine-Drools-flow			workflow engines → clinicians.
Yılmaz Ö, Erdur RC, Türksever M, 2013 [95]	SWRL	Protégé-OWL	Guidelines	Jess	-	-	Patient data + rule + knowledge → recommendation
Bau CT, Chen RC, Huang CY, 2014 [96]	Jena	Jena Rules	Disease, management, patient	Jena inference engine	OWL-DL, Protégé, Concept Explorer (ConExp), Jena rules	-	Patient data + patient ontology + Jena rules (from knowledge base) → IENA inference engine → recommendation
Gallerani M, Pelizzola D, Pivanti M, et al, 2014 [99]	XML	-	Publications, local domain experts	Drools rule engine	-	HL7	User request → patient data + application server check → recommendations + archive
Goldberg HS, Paterno MD, Rocha BH, et al, 2014 [27]	XML	-	Guidelines	Operational decision manager (ODM) JBoss	-	HL7 CCD	Patient data + rules → recommendations
Sesen MB, Peake MD, Banares-Alcantara R, et al, 2014 [98]	OWL	-	NICE, BTS, ESMO, NCCN guidelines, Bayesian network from existing databases	-	Not specified	-	-
Stewart SA, Abidi S,	SWRL	-	Recommendations	-	-	-	CPG computerization → CPG execution + patient profiles →

Parker L, et al, 2014 [100]							personalized diary composition/ recommendation
Robles-Bykbaeva V, López-Noresb M, Pazos-Ariasb J, et al, 2015 [104]	Archetype	OpenEHR	Guidelines	-	-	-	-
Abidi SR, Cox J, Abusharekh A, et al, 2016 [106]	SWRL rules	-	Canadian clinical guidelines for atrial fibrillation	CPG execution engine	-	-	NOAC ontology + patient data → recommendation via auto-fill PDF
Goldberg HS, Paterno MD, Grundmeier RW, et al, 2016 [108]	XML	Operational Decision Manager (OPM)	Literature, prior trials	-	Production rules	HL7	Patient data + prediction rules → recommendations
Zhang YF, Tian Y, Zhou TS, et al, 2016 [107]	Jena	Protégé + Jena semantic framework	Clinical guidelines, care plans, domain experts, EMR	Jena inference API	Jena rule	HL7 CDA as input and output	Patient data + ontology + rules → inference API → HL7 CDA → EMR
Abidi S, 2017 [114]	OWL-DL	Protégé	Clinical practice guidelines, domain experts; drug management protocols; expert-led reconciliation of CPGs	Reasoning engine	-	-	CPG ontology → computable CPG → comorbid CPG knowledge model → patient data + physician input → recommendation
Chen RC, Jiang HQ, Huang CY, et al, 2017 [112]	Jena rules, fuzzy rules	-	Guidelines, Technique for Order of Preference by Similarity to Ideal	Jena inference engine	-	-	Patient data + fuzzy rules + drug knowledge base → recommendations

			Solution (TOPSIS)				
Shang Y, Wang Y, Gou L, et al, 2017 [111]	Jena	Rules are within the knowledge base, Protégé	CPG knowledge class, coding system class, patient class, disease, factors, treatment, recommendation	Jena reasoner + SPARQL	Java, Jena API, Jena rules	HL7	Patient data + knowledge query + rules → rule engine → recommendation
Zhang YF, Gou L, Zhou TS, et al, 2017 [110]	Jena rules	Protégé	Clinical guidelines, nurses	-	-	HL7 and the Object Management Group (OMG)	Domain knowledge + patient data → assessment criteria → recommendation
Nakawala H, Ferrigno G, De Momi E, 2018 [116]	SWRL, OWL-DL	Protégé	Textbooks, interviews, web resources	-	-	-	Surgical procedures knowledge; instrument recognition
Séroussi B, Guézennec G, Lamy JB, et al, 2018 [117]	Formal module-driven rule language (NRL), N3, XML	-	PubMed, NCCN, ESMO, SEOM Clinical guidelines	-	N3	FHIR to exchange input and output of CDS	DSS query FHIR server → +patient data → FHIR resources + rules + inference engine → recommendations
Séroussi B, Lamy JB, Muro N, et al, 2018 [119]	N3	-	Clinical guidelines, experience, prior cases	-	-	FHIR	Patient data → FHIR message → reasoning ontology → recommendations
El-Sappagh S, Ali F, Hendawi A, et al, 2019 [124]	SWRL	Protégé 5.1 editor	CPG, domain experts, web	Rule-based reasoner (e.g., Pellet)	Protégé, Jena API	Knowledgebase incorporated HL7 FHIR standards	Patient data + medical knowledge → reasoner → recommendation
Jafarpour B, Raza Abidi S,	OWL2	-	Guidelines	Jena reasoner	-	-	Clinical state change + external events + rules + ontology →

Van Woensel W, et al, 2019 [123]							integration engine → recommendation
Nguyen BP, Reese T, Decker S, et al, 2019 [29]	CQL	-	Decision trees, guidelines	CQL engine	-	FHIR	Patient data + event → CQL library check → alerts or recommendations
Semenov I, Osenev R, Gerasimov S, et al, 2019 [122]	-	Rule editor has an interface, no name	Clinical guidelines	Rule engine and Bayes engine	-	FHIR JSON	-
Román-Villarán E, Pérez-Leon FP, Escobar-Rodriguez GA, et al, 2019 [121]	OWL	-	Clinical guidelines, healthcare professionals	-	OWL API	HL7 FHIR	Patient data + CPG + prior treatment data of the patient's → recommended treatment plan, personalized information
Maldonado JA, Marcos M, Fernández-Breis JT, et al [125]	OWL, Archetype		Cancer screening protocols (USA, European)	Inference reasoning		Transforming, Semantic publishing, Mapping	

Abbreviations: -, not specified; ADA: American Diabetes Association; ADE: adverse drug events; AF: atrial affiliation; API: application programming interface; BTS: British Thoracic Society; CCD: continuity of care document; CDA: clinical document architecture; CDSS: clinical decision support system; CIG: computer interpretable guidelines; CLIPS: C Language Integrated Production System; COPD: chronic obstructive pulmonary disease; CPG: clinical practice guidelines; CQL: clinical quality language; DL: description logic; EMR: electronic medical records; ESMO: European Society for Medical Oncology; FDA: Food and Drug Administration; FHIR: Fast Healthcare Interoperability Resources; HIS: hospital information systems; HL7: health level 7; JESS: Jave Expert System Shell; MLM: medical logic module; NCCN: National Comprehensive Cancer Network; NICE: National Institute for Clinical Excellence; NOACs: New Oral Anticoagulants; NRL: natural rule language; OWL: W3C Web Ontology Language; RIM: reference information model; SPARQL: SPARQL protocol and RDF query language; SQWRL: a query language for OWL; SWRL: Semantic Web Rule Language; UMLS: unified medical language system; WHO: World Health Organization; XML: extensible markup language.
