# Supplementary material for: Ontologies Applied in Clinical Decision Support System Rules: Systematic Review
Source: JMIR Med Inform. 2023 Jan 19;11:e43053. doi: 10.2196/43053 (PMC9896360; doi:10.2196/43053)
Supplement: Multimedia Appendix 10 [file medinform_v11i1e43053_app10.pdf]

Appendix 10 Comparison of ontology roles in included publications (*n* = 36)

**Authors/ year**	**Ontology is used for**	**Specify CDSS rules/general knowledge**	**Validated by domain experts**	**Reasoner**	**Ontology source/language**	**With CDSS**	**With EMR/HIS**
De Clercq PA, Blom JA, Hasman A. et al, 2000 [53]	Knowledge source	Specify CDSS rules	Yes	-	-	-	-
Achour SL, Dojat M, Rieux C, 2001 [55]	Build MLM	Specify CDSS rules	Created by domain experts	Not specified	UMLS	Communicate	With prototype/HIS
Liaw ST, Sulaiman N, Pearce C, et al, 2003 [60]	-	-	Yes	-	ICD10-AM, ICPC2	-	HL7
Kashyap V, Morales A, Hongsermeier T, 2006 [63]	Classifier	Both	Yes, by knowledge base authors	OWL reasoner-Cerebra	Separate - ILOG	Communicate	Services on clinical data repository
Abidi SR, 2007 [65]	Knowledge source for recommendations	None specify/ Guidelines in GEM	Consult oncologist, individual clinical experience	-	Guidelines/OWL	GEM execution engine	-
Carenini M, 2009 [73]	Classify alerts and notifications	-	Experts validate taxonomy	-	JCAHO patient safety event taxonomy, ICPS, DOLCE	-	-
Farion K, Michalowski W, Wilk S, et al, 2009 [70]	Derive data, support, interface, configuration models	Both	Yes	-	-	Support ontology, executor	Implemented in prototype, web services

Ongenae F, Dhaene T, De Turck F, et al, 2010 [75]	Classify temporal patterns	Both	Yes	DL reasoner-Pellet, probabilistic reasoner-Pronto	Rule-based reasoner	Decision module	New patients added to the ontology → automatically classified
Bouamrane MM, Rector A, Hurrell M, 2011 [78]	Classifications, recommendation	Both	Yes	Java-based Pellet	OPCS, ICD-10, NICE guideline/OWL	-	OWL, OWL API developed the system
Cao F, Sun X, Wang X, et al, 2011 [80]	Reasoning to get implicit relationships	Both	-	TBox and ABox reasoning over SNOMED	SNOMED-CT	Ontology mapping to ADE repository	Deployed in hospital as a Web application
Lee CS, Wang MH, 2011 [81]	-	Specify CDSS rules	Yes	-	Internet/OWL	-	-
Bright TJ, Yoko Furuya E, Kuperman GJ, et al, 2012 [83]	Generate prescribing alerts	Both	Yes	Jess rule engine	-	Alert module	Protégé + patient data + SWRL tab
Grando A, Farrish S, Boyd C, et al, 2012 [85]	Safe and effective polypharmacy prescription	Both	Yes	Jess reasoner	RxNorm, UMLS, COPD, hypertension, osteoporosis, diabetes guidelines	Conceptual test	Conceptual test with cases
Chniti A, Boussadi A, Degoulet P, et al, 2012 [86]	Author, execute, and manage business rules	Both	Yes	Java-based Jena	JRules OWL plug-in	Not integrated with HIS	CDS rules were implemented in HIS
Riaño D, Real F, López-Vallverdú JA,	Classification cases and treatment plans	Specify CDSS rules	Health care professionals provided some content	Jena	ICD10 CM, ATC/SDA modeling language	-	-

et al, 2012 [82]							
Artetxe A, Sanchez E, Toro C, et al, 2013 [89]	Infer clinical tests on patients to detect Alzheimer’s disease	Both	Authored by domain experts	None specified; reasoning module	SNOMED CT, SWAN	The testing system of CDSS	Large scale Spanish research project
Sáez C, Bresó A, Vicente J, et al, 2013 [91]	Inference recommendation, assessment	Both	-	Rule-based reasoner	SNOMED CT	Case study test in a rule-based decision support module	Integrated with a telemedicine health platform
Yao W, Kumar A, 2013 [88]	Infer context information, patient conditions, rules for CDSS	Both	-	Pellet reasoner plug-in for Protégé 3.4	Jess rule engine, workflow engine-Drools-Flow 5.2	Scenario test, integration with clinical workflows	CONFlexFlow implementation, web-based services
Yılmaz Ö, Erdur RC, Türksever M, 2013 [95]	Inference new facts	Both	-	-	ICD/OWL	-	-
Bau CT, Chen RC, Huang CY, 2014 [96]	Infer recommendations for patients	Both	Yes	Jena	Jena inference engine	CDSS prototype	Patient data + CDSS
Sesen MB, Peake MD, Banares-Alcantara R, 2014 [98]	Ontological guideline rule inference	Specify CDSS rules	-	FaCT++	NICE Guidelines, OWL, mapping to SNOMED CT	OWL API + FaCT ++ semantic reasoning + probabilistic reasoning	JDBC connect to patient records database
Stewart SA, Abidi S, Parker L, et al, 2014 [100]	Computerized CPG	Specify CDSS rules	Domain experts evaluate rules	Pellet reasoner	CPG, OWL-DL	-	Embedded in an EHR portal in Canada

Abidi SR, Cox J, Abusharekh A, et al, 2016 [106]	Knowledge sources, criteria	Specify CDSS rules		OWL reasoning	AF clinical practice guidelines/OWL	Computerized NOAC authorization decision support system	Can be integrated with EHR or stand-alone web version
Marco-Ruiz L, Pedrinaci C, Maldonado JA, et al, 2016 [109]	Describing CDS services, functional, nonfunctional, and data semantics	Both	Use case validation	OWL Horst	SNOMED CT, Dublin Core/OWL	-	-
Zhang YF, Tian Y, Zhou TS, et al, 2016 [107]	Infer diagnosis, recommendations	Both	Yes /examined by domain experts	Pellet	An inference API – Jena rules	Experiments on CDSS	CDA format used to convert between EMR and CDSS
Abidi S, 2017 [114]	Knowledge sources	Specify CDSS rules	Yes, via evaluation by independent cardiologists, PCP	Pellet	Practice guidelines, domain experts; protocols	Communicate	Stand-alone EMR
Shang Y, Wang Y, Gou L, et al, 2017 [111]	Infer personalized recommendations	Both	Yes	None specify	Jena API	Web services for CDSS	Web API by using patient data + CDSS
Zhang YF, Gou L, Zhou TS, et al, 2017 [110]	-	-	-	Pellet	SNOMED CT /OWL-DL	-	EMR and other sources
Nakawala H, Ferrigno G, De Momi E, 2018 [116]	Knowledge sources	Specify production rules	Yes	Pellet	Journal article, physician, online resources, HONcode search engine; FMA, IAO, BFO, W3C time ontology	-	-

Séroussi B, Guézennec G, Lamy JB, et al, 2018 [117]	Knowledge sources	Specify CDSS rules	-	Euler/EYE inference engine	NCI Thesaurus, LOINC, SNOMED CT, OWL	-	-
Séroussi B, Lamy JB, Muro N, et al, 2018 [119]	Knowledge sources	Specify CDSS rules	-	Euler/EYE inference engine	Guidelines	Web services on CDSS	-
Shen Y, Yuan K, Chen D, et al, 2018 [115]	Identify disease and recommend treatment	Both	-	-	IDO, NCBI Taxonomy, HPO, DrugBank, DO, Internet, Guidelines	Clinical decision module	Patient data + ontology + clinical decision moduel + user interaction module
El-Sappagh S, Ali F, Hendawi A, et al, 2019 [124]	Provide a personalized care plan	Both	Yes	Pellet	SWRL rule engine, Jena API	Cloud-based CDSS	FHIR patient data + CDSS + ontology → FHIR → EHR
Jafarpour B, Raza Abidi S, Van Woensel W, et al, 2019 [123]	Provide domain knowledge and integration policies	-	A medical expert validated the integration solution; medical experts instantiate integration policies	Jena reasoner	SNOMED, Drug Bank/ OWL	-	Integration framework with web API, CIG server and client, client connect POC
Román-Villarán E, Pérez-Leon FP, Escobar-Rodriguez GA, et al, 2019 [121]	-	Specify CDSS Rules	Yes, by clinical researchers	-	Mapped to SNOMED CT	-	-

Maldonado JA, Marcos M, Fernández-Breis JT, et al [125]	Classification of risk level	Both	-	OWL reasoner	LOINC	Web-based platform	Patient data from EHR + platform

Abbreviations: -, not specified; AD: Alzheimer's disease; ADE: adverse drug events; AF: atrial affiliation; API: application programming interface; BFO, Basic Formal Ontology; CDA: clinical document architecture; CDSS: clinical decision support systems; CIG: computer interpretable guidelines; COPD: chronic obstructive pulmonary disease; COSI: closed world OWL interpreter; CPG: clinical practice guidelines; DL: description logic; DO: disease ontology; DOLCE: Descriptive Ontology for Linguistic and Cognitive Engineering; EHR: electronic health records; EMR: electronic medical record; FCA: formal concept analysis; FHIR, HL7 fast healthcare interoperability resources; FMA, Foundational Model of Anatomy; GEM: guideline elements model; HIS: hospital information system; HL7: health level 7; HPO: Human phenotype ontology; IAO, Information Artifact Ontology; ICD10-AM: the International Statistical Classification of Diseases and Related Health Problems, Tenth Revision, Australian Modification; ICPC: International Classification of Primary Care; ICPS: International Classification for Patient Safety; IDO: infectious disease ontology; JCAHO: the Joint Commission on Accreditation of Healthcare Organizations; MLM: medical logic module; NCBI: National Center for Biotechnology Information; NICE: National Institute for Clinical Excellence; NOACs: New Oral Anticoagulants; OWL: Web Ontology Language; OPCS: Office of Population Censuses and Surveys; PCP: primary care providers; POC: point of care; SDA, state-decision-action; SNOMED-CT: Systemized Nomenclature of Medicine – Clinical Terms; SWAN: Semantic Web Applications in Neuromedicine; SWRL: Semantic Web Rule Language; UMLS: unified medical language system.
